# Quantitative single cell analysis uncovers the life/death decision in CD95 network

**DOI:** 10.1371/journal.pcbi.1006368

**Published:** 2018-09-26

**Authors:** Jörn H. Buchbinder, Dennis Pischel, Kai Sundmacher, Robert J. Flassig, Inna N. Lavrik

**Affiliations:** 1 Translational Inflammation Research, Otto von Guericke University, Magdeburg, Germany; 2 Process Systems Engineering, Otto von Guericke University, Magdeburg, Germany; 3 Process Systems Engineering, Max Planck Institute for Dynamics of Complex Technical Systems, Magdeburg, Germany; 4 The Federal Research Center Institute of Cytology and Genetics, The Siberian Branch of the Russian Academy of Sciences, Novosibirsk, Russia; Ecole Normale Supérieure, FRANCE

## Abstract

CD95/Fas/APO-1 is a member of the death receptor family that triggers apoptotic and anti-apoptotic responses in particular, NF-κB. These responses are characterized by a strong heterogeneity within a population of cells. To determine how the cell decides between life and death we developed a computational model supported by imaging flow cytometry analysis of CD95 signaling. Here we show that CD95 stimulation leads to the induction of caspase and NF-κB pathways simultaneously in one cell. The related life/death decision strictly depends on cell-to-cell variability in the formation of the death-inducing complex (DISC) on one side (extrinsic noise) *vs*. stochastic gene expression of the NF-κB pathway on the other side (intrinsic noise). Moreover, our analysis has uncovered that the stochasticity in apoptosis and NF-kB pathways leads not only to survival or death of a cell, but also causes a third type of response to CD95 stimulation that we termed ambivalent response. Cells in the ambivalent state can undergo cell death or survive which was subsequently validated by experiments. Taken together, we have uncovered how these two competing pathways control the fate of a cell, which in turn plays an important role for development of anti-cancer therapies.

## Author summary

Activation of death receptor (DR) family has been reported to activate both apoptotic as well as anti-apoptotic responses. Molecular mechanisms underlying the intricate details of this crosstalk have not been established yet. Here we show that these pathways are triggered simultaneously in one cell. Furthermore, using stochastic computational modeling we uncovered how an individual cell undergoes apoptosis, while other cells survive upon the same DR activation conditions. This was only possible by combination of computational modeling supported by experimental validation based on the state of the art single cell analysis. The latter included cutting edge technology of imaging flow cytometry, which combines microscopy and flow cytometry in one measurement circuit enabling quantitative analysis of endogenous cellular protein levels estimated from a large number of cells simultaneously. This allowed to shed the light on the question how a single cell possibly avoids apoptosis, which is a highly actual topic in the field of cancer research and development of efficient anti-cancer therapies.

## Introduction

Apoptosis is a program of cell death, which is essential for all multicellular organisms [1]. The crosstalk between apoptotic and anti-apoptotic pathways plays a key role in shaping life/death decisions in the cell. Furthermore, the success of anti-cancer therapies strongly depends on the efficiency of cell death induction in a single cell. However, a number of apoptotic stimuli are well known to activate strong anti-apoptotic response, which naturally might prevent apoptosis by upregulation of anti-apoptotic genes and, hence, counteract the effect of anti-cancer therapies [2,3].

In particular, members of the death receptor (DR) family have been reported to activate both apoptotic as well as anti-apoptotic responses [1,4]. The DR family is a subfamily of TNFR-superfamily, which includes TNF-R1, CD95/Fas and TRAIL-R1/2 [5]. Triggering of the DR family with the cognate death ligands results in the formation of high molecular weight complexes, which induce cell death pathways and anti-apoptotic responses including activation of the transcription factor NF-κB [6–9]. The induction of a particular pathway in the DR system is a rather complex process, which is highly context dependent and multifactorial [8,10]. Importantly, molecular mechanisms underlying the intricate details of the cross-talk between apoptotic and anti-apoptotic pathways have not been established yet.

The complexity of the response to DR activation stems from the fact that it is often heterogeneous within a population of cells [11–13]. The sources of the heterogeneity include genetic variations within cells, cell cycle effects, and stochastic effects from gene translation/transcription, which cumulatively might lead to different initial abundances of proteins within different cells [10,13]. For deciphering the multifactorial nature of cell death decisions in single cells, computational modeling paired with new experimental technologies providing a large number of data for the protein expression levels from individual cells are of indispensable value [12,14–16]. Of particular importance in this regard, is the cutting edge technology of imaging flow cytometry (IFC), which combines microscopy and flow cytometry in one measurement enabling quantitative analysis of endogenous cellular protein levels estimated from a large number of cells simultaneously [17,18]. This feature of IFC is a strong advantage compared to confocal imaging that is mostly based on the application of artificially overexpressed activity probes, which might be often misleading. Furthermore, IFC in combination with machine learning provides a unique platform to quantitatively assign single cell events over a large number of cells and thereby has a strong advantage over confocal microscopy, which often needs manual analysis.

In this study we addressed the interplay of apoptotic and anti-apoptotic pathways in single cells by analyzing the signaling network of the exemplified member of the DR family CD95. CD95 stimulation leads to formation of the CD95 death-inducing signaling complex (DISC) [1]. The DISC comprises CD95, the adaptor protein FADD, the initiator procaspase-8a/b (p55/p53), procaspase-10 and c-FLIP (S1A Fig). After recruitment to the DISC, procaspase-8 builds death effector domain (DED) chains/filaments, formed *via* homotypic interactions between the DEDs of individual procaspase-8 molecules [19–21]. This provides the platform for homodimerization of procaspase-8 molecules, subsequent activation of procaspase-8 homodimers, and their processing with formation of the active caspase-8 heterotetramers p10_2_-p18_2_ (S1A Fig). The activation of caspase-8 might be blocked by c-FLIP proteins, which incorporate into the DED chains and form heterodimers with procaspase-8 [21–23]. Interestingly, c-FLIP proteins act not only as inhibitors of CD95-induced apoptosis but are also essential for NF-κB activation [22,24]. Namely, the c-FLIP cleavage products p43-FLIP and p22-FLIP (S1A Fig), have been reported to induce NF-κB [24–26]. The p43-FLIP cleavage product has been shown to play an essential role for CD95-mediated NF-κB activation [22]. The classical NF-κB activation pathway involves degradation of the NF-κB bound inhibitors of kappa B (IκBs), which are phosphorylated by the IκB kinase (IKK)-complex containing two catalytic subunits (IKKα and IKKβ) and the regulatory subunit NF-κB essential modulator (NEMO). Consequently, the NF-κB dimer (p65/RelA and p50) is released to enter the nucleus, where activation of the transcription of the target genes takes place [27].

The intricate regulation of cell fate upon induction of CD95-mediated apoptosis *vs*. NF-κB at the single cell level remains largely unknown. Recently, a number of studies have highlighted the importance of the caspase-8 activation rate in single cells for the initiation of CD95-mediated apoptosis or survival [12,16]. However, the role of CD95-induced crosstalk of apoptosis with the anti-apoptotic signaling pathways such as NF-κB in the cell fate has so far only been addressed at the population level and never at the single cell level [24,28]. In this study we have investigated the regulation of these competing pathways in single cells using computational modeling, cutting edge technology of IFC and quantitative western blot. We show that cell-to-cell variability in the apoptotic phenotype does not result from NF-κB related stochasticity, but from heterogeneities in the composition of DED chains at the CD95 DISC.

## Results

### CD95 stimulation simultaneously induces apoptosis and NF-κB in one cell

**Fig 1:**
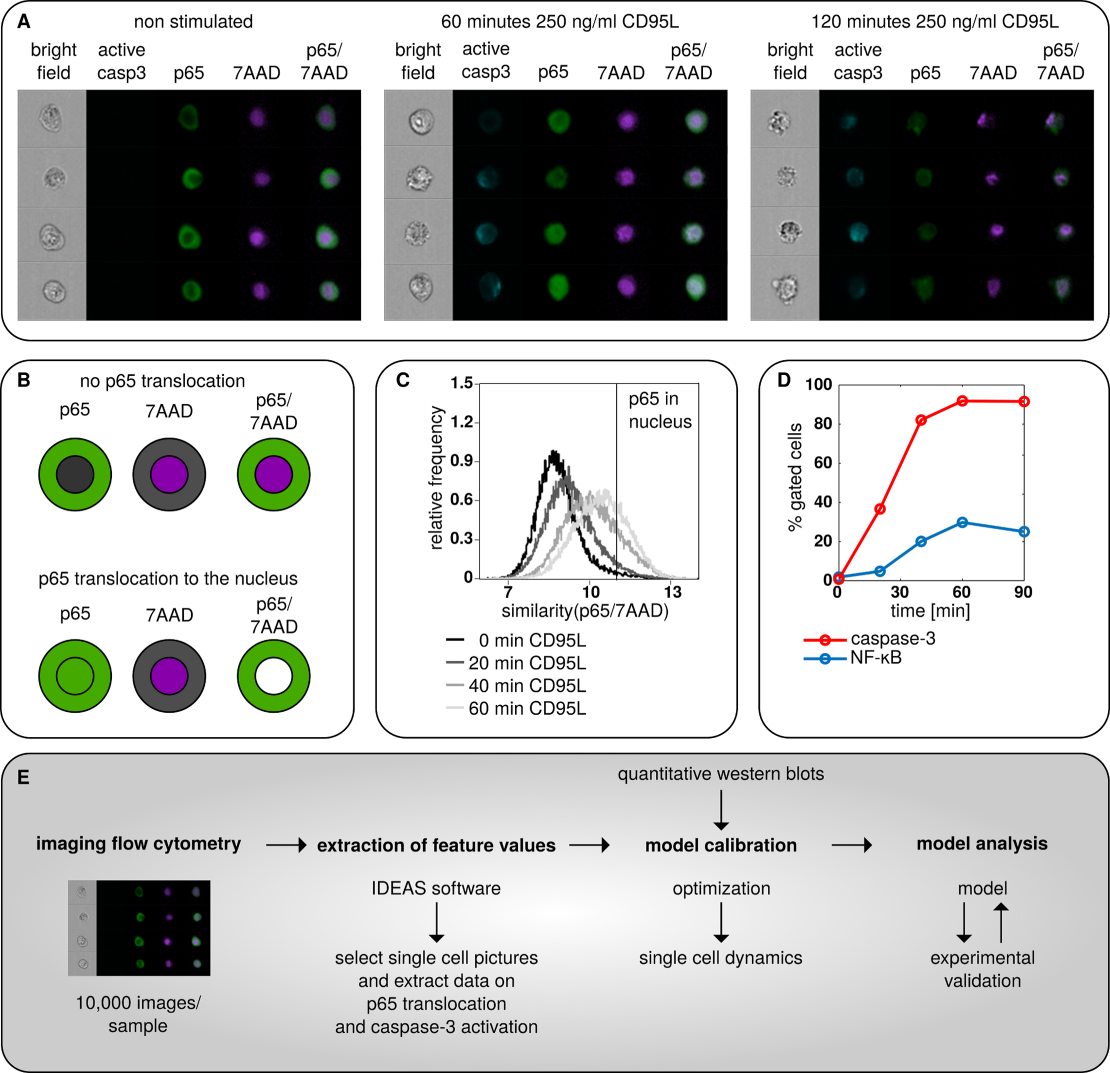
CD95 stimulation induces caspase-3 and NF-κB activation. (A) HeLa-CD95 cells were stimulated with 250 ng/ml of CD95L for indicated time intervals; and stained with antibodies against the NF-κB subunit p65 and active caspase-3 as well as 7AAD. Images acquired by Amnis FlowSight: bright field images, active caspase-3 (light blue), p65 (green), 7AAD (purple), and an overlay of p65 and 7AAD are shown for four independent cells for each condition to illustrate the technique. (B) Scheme of p65 (green) translocation to the nucleus (purple). Cells with p65 translocation are displayed with nucleus depicted in white, which results from overlaying the green p65 and purple nuclear 7AAD signal. (C) Representative diagram of similarity score between nucleus and p65 for indicated CD95L stimulation intervals. The similarity score for p65 translocation to the nucleus taken as a threshold value is shown with a black vertical line. (D) Abundance of cells with nuclear NF-κB translocation and caspase-3 activation. (E) Workflow of a study demonstrating connections between experimental and computational analysis.

Previously, it has been demonstrated that stimulation of CD95 with CD95L induces activation of both apoptotic and NF-κB pathways [6,9,24]. Several reports suggested that upon DR stimulation, in particular CD95, some cells undergo apoptosis while other cells solely induce NF-κB [6]. Western blot analysis, as a “bulk population” measurement, does not allow to distinguish whether caspase activation and NF-κB induction occur in the same or distinct cells (S1B Fig). To address this question, we used IFC, which allows to quantitatively analyze signaling events in a large number of single cells and to quantify these events at the population level. In particular, we monitored caspase-3 activation and p65 translocation to the nucleus and quantified them over 10,000 cells as described before [18]. HeLa cells overexpressing CD95 (HeLa-CD95 cells) were stimulated with 250 ng/ml CD95L followed by immunostaining with anti-p65 and anti-active caspase-3 antibodies in combination with staining of the nucleus with the DNA dye 7AAD (Fig 1A and 1B). p65 translocation to the nucleus is a well described feature of NF-κB activation [29,30]. Accordingly, the similarity of the p65 and 7AAD signals in the nucleus serves as readout for NF-κB activation in IFC, which was also used in our study (Fig 1B and 1C, S2A Fig). Furthermore, the timing of p65 translocation to the nucleus was consistent with a time course of p65 phosphorylation at Ser536 as well as degradation and phosphorylation of IκBα, which further verifies this approach of measuring NF-κB activation in single cells (S1B and S1D Fig, S2B and S2C Fig).

In accordance with our previous report [18] we have observed that CD95 stimulation results in the detection of four populations of cells: the first population does not show caspase-3 or p65 nuclear translocation. In the second one, only cells with p65-nuclear translocation while in the third population cells with active caspase-3 were observed. Finally, the fourth population consisted of cells that were characterized by activation of both pathways: apoptosis and NF-kB as manifested by both active caspase-3 and nuclear p65 detection. In this way, we observed that CD95 stimulation of HeLa-CD95 cells led to the appearance of nuclear p65 and active caspase-3 in the same cells (Fig 1D). Furthermore, the response to CD95L stimulation was rather heterogenic: some cells showed stronger caspase-3 and NF-κB activation than others. This raised the question of how this heterogeneity manifests in life *vs*. death decisions and how it affects the dynamics of regulation of these two competing signaling pathways. To understand these processes and possibly delineate new molecular mechanisms beyond these distinct responses we applied computational modeling (Fig 1E).

### The CD95 network model

**Fig 2:**
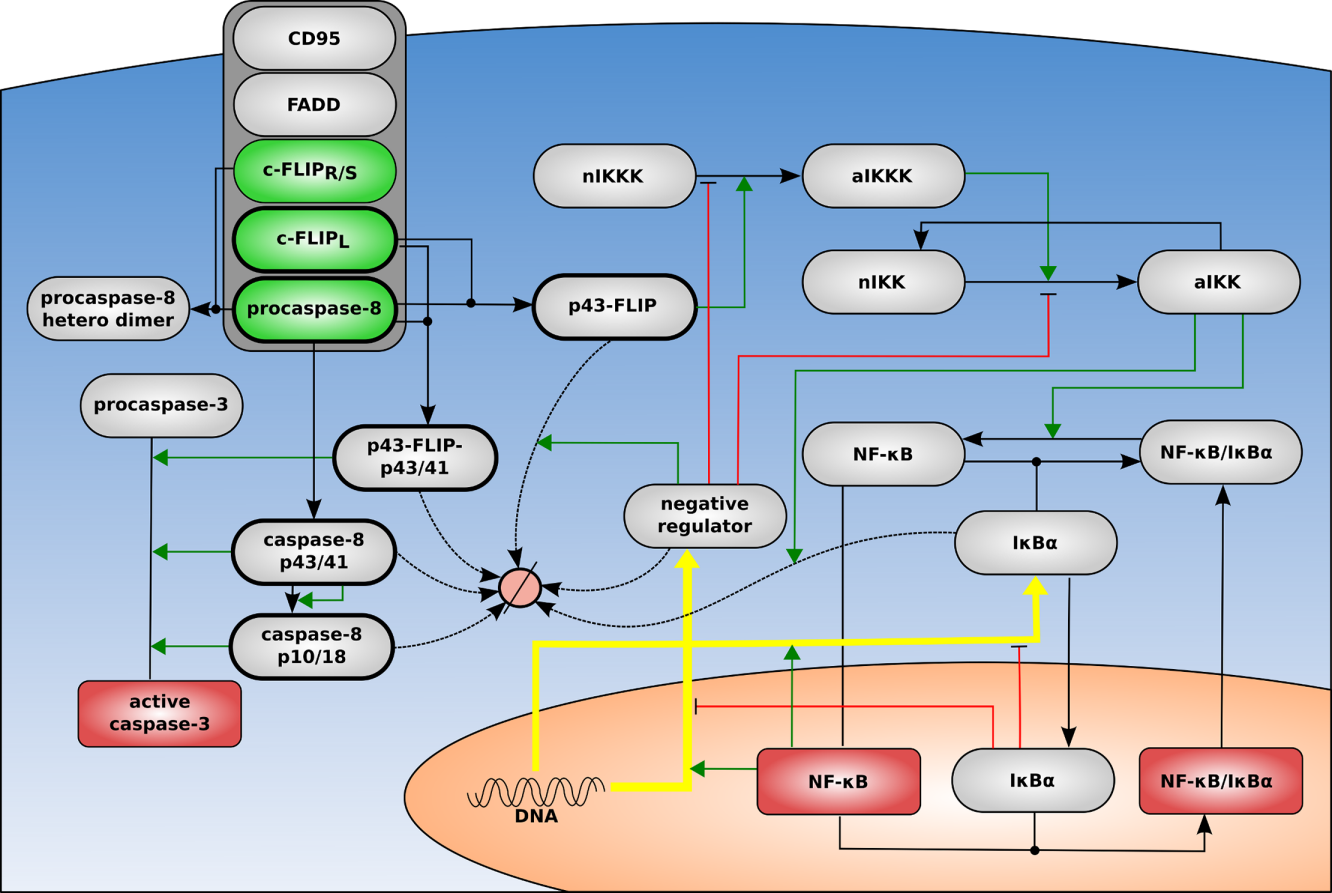
Model topology. The model of CD95 network comprises the cytosol (blue), surrounded by the cell membrane, and the nucleus (orange). The subnet of this model is CD95 DISC (gray). The CD95 DISC comprises CD95, FADD, and DED chains/filaments. Procaspase-8, c-FLIP_L_, and c-FLIP_R+S_ contribute to the DED chain/filament formation and embody the source of extrinsic noise (green). In contrast intrinsic noise is introduced by stochastic gene expression (yellow arrows). The relative amounts of procaspase-8, its cleavage products, and c-FLIP proteins were measured by western blot (thick frame), while active caspase-3 and nuclear NF-κB by imaging flow cytometry (red). Inhibitions are marked by red lines and activations by green arrows.

The CD95 signal transduction network implemented in the model is initiated by the CD95 DISC leading to induction of apoptotic and NF-κB pathways [19,23] (Fig 2). After DISC assembly and DED chain formation, resulting in procaspase-8 activation and cleavage to p43/p41, caspase-3 is activated, which leads to apoptosis (Fig 2). For this part we used a simplified version of the model introduced by Fricker and colleagues [31]. The topology of the anti-apoptotic pathway in the model was constructed in accordance with previous reports [24]. The c-FLIP_L_ cleavage product p43-FLIP generated in the DED chain by procaspase-8 provides a link to the induction of the NF-κB pathway *via* activation of IKK complex that enforces phosphorylation and degradation of IκBα (Fig 2). IκBα binds NF-κB in the cytosol and thereby regulates the access of NF-κB to the nucleus. After degradation of IκBα, NF-κB enters the nucleus and induces expression of its target genes. The modeling of nuclear events following NF-κB activation was simplified. In our model only the synthesis of IκBα that keeps NF-κB in the cytosol, and the generation of a so-called generic negative regulator that deactivates IKKK and IKK, have been introduced. This part of the model strongly implements the topology of a previously introduced NF-κB model [32]. Taken together, in the constructed model active caspase-3 serves as a link between caspase-8 activation in the DED chain and apoptosis execution, while p43-FLIP generated in the DED chain connects the latter to the NF-κB pathway. In this way, the DED chain in our model gives rise to both caspase-3 and NF-κB activation promoting both pathways simultaneously.

**Fig 3:**
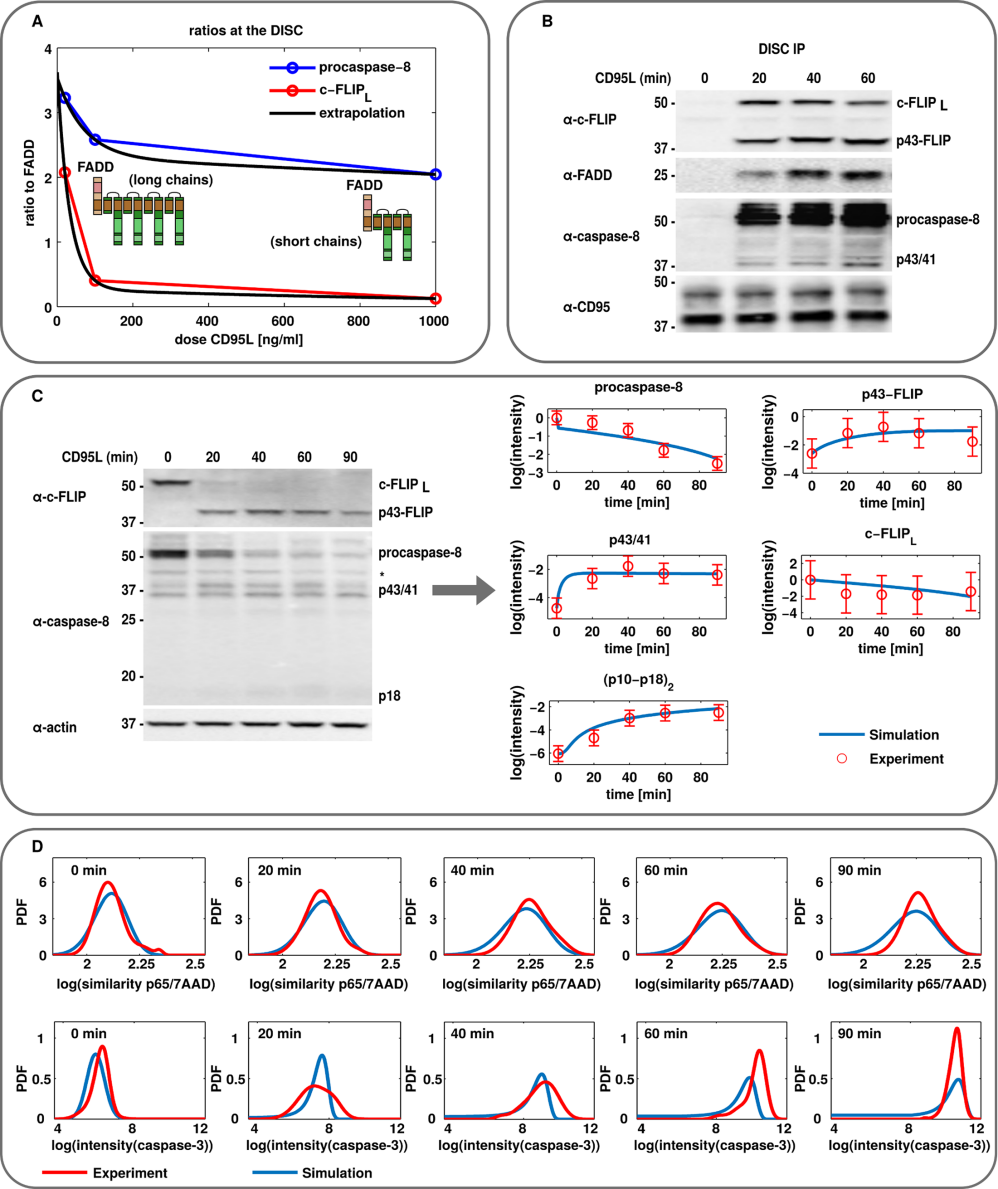
Quantification of experimental data of CD95 signaling. (A) The experimental data on c-FLIP (red) and procaspase-8 ratios to FADD (blue), respectively, were taken from Schleich et al. [20]. The corresponding extrapolation of these ratios for HeLa-CD95 cells (black) describes the chain configuration dependency on the CD95L stimulation dose. (B) Western blot analysis of DISC immunoprecipitation (IP) from HeLa-CD95 cells stimulated with 250 ng/ml CD95L for indicated time intervals. See S3 Fig for more details. (C) Representative example of application of quantitative Western blot data for modeling. Western blot analysis of signaling in HeLa-CD95 cells stimulated as in (B). One out of three representative experiments is shown (Complete data set is presented in S4 Fig). The model predictions (blue) are compared to the experimentally measured values (red). Means and SDs are shown. (D) Representative example of imaging flow cytometry data application for modeling. HeLa-CD95 cells were stimulated with 250 ng/ml CD95L and stained with antibodies against NF-κB subunit p65 and active caspase-3. The nucleus was stained with 7AAD. The probability density function (PDF) of the similarity score of the p65 and nuclear signal as well as the intensity of the caspase-3 signal were analyzed (red) and compared to the model predictions (blue). * = nonspecific band.

The DISC was modeled as a subnet and, subsequently, a submodel of the global CD95 network/model (Fig 2). The formation of three types of procaspase-8 homo- and heterodimers in the DED chain was introduced in the model: procaspase-8 homodimer, procaspase-8/c-FLIP_L_ heterodimer, and procaspase-8/c-FLIP_S_ heterodimer [23,31,33]. The first two dimers were catalytically active and underwent processing, while the procaspase-8 heterodimer with c-FLIP_S_ was catalytically inactive (Fig 2). Furthermore, in order to simplify the modeling of the DED chain assembly, only ratios of DED proteins but not their variable positions in the chain were taken into account for the modeling. To define the ratio of procaspase-8/c-FLIP in the DED chain, their ratios were estimated in HeLa-CD95 cells *via* extrapolation from the corresponding ratios in SKW6.4 cells upon different stimulation strength (Fig 3A). Consistent with previous reports, we also considered that strong stimulation results in shorter chains, while weak stimulation results in longer chains with a higher abundance of c-FLIP [20,22] (Fig 3A). The model in SBML form is provided in the S1 Appendix.

### Sources of heterogeneity in the CD95 model: Extrinsic and intrinsic noise

To account for the heterogeneity of the response to CD95 stimulation, *e*.*g*. the variabilities in the strength of caspase-3 *vs*. NF-κB activation, we used a stochastic modeling approach. Two sources of variability were incorporated into the model: extrinsic stochasticity, which was considered to arise from cell-to-cell variability in protein levels and intrinsic stochasticity resulting from different activities of the single components of intracellular signaling pathways arising from probabilistic processes of interactions between individual molecules. Since our knowledge regarding the initial distribution of all chemical species is limited to the proteins responsible for the DISC formation only procaspase-8, c-FLIP_L_ and c-FLIP_S_ contribute to extrinsic noise in our model. We assume that these proteins obey a log-normal distribution with a standard deviation proportional to the mean. The proportionality factor was derived from data provided in [31]. It is known that intrinsic noise is very dominant when only a few copy numbers of a chemical species are present. Hence, the switching of genes from the active to the inactive state and the corresponding backward reactions are modeled as stochastic processes. In contrast the remaining reactions are treated deterministically.

The generated model was trained against a merged set of single cell and population-level data, as indicated in the modeling workflow in Fig 1E. Single cell data were obtained *via* IFC analysis of caspase-3 activation and p65 nuclear translocation for stimulation strength from 10 to 250 ng/ml CD95L and for time intervals from 20 to 90 minutes (Fig 3D, S3–S6 Figs, S1 Text). To broaden the data set for estimation of the model parameters, quantitative western blot analysis was performed for the analysis of signaling in HeLa-CD95 cells stimulated with 250 ng/ml CD95L (Fig 3B and 3C, S3 and S4 Figs, S1 Text). This allowed measuring time-dependent processing of procaspase-8 to p43/p41 and p18 as well as the cleavage of c-FLIP_L_ to p43-FLIP on the population level. Dynamic single cell and population data were integrated into the model by distinct approaches (S1 Text). It turned out that the dynamics of the calibrated model are able to qualitatively capture the experimental data (Fig 3C and 3D, S1 Text). We then used the calibrated model for subsequent analysis to compute the temporal evolution of single cell states *via* a hybrid version of the Gillespie algorithm accounting for the intrinsic noise [34] and Monte Carlo sampling of the initial conditions to compute extrinsic noise.

### Model analysis uncovers dose-dependent caspase activation and robustness in CD95-mediated NF-κB activation

**Fig 4:**
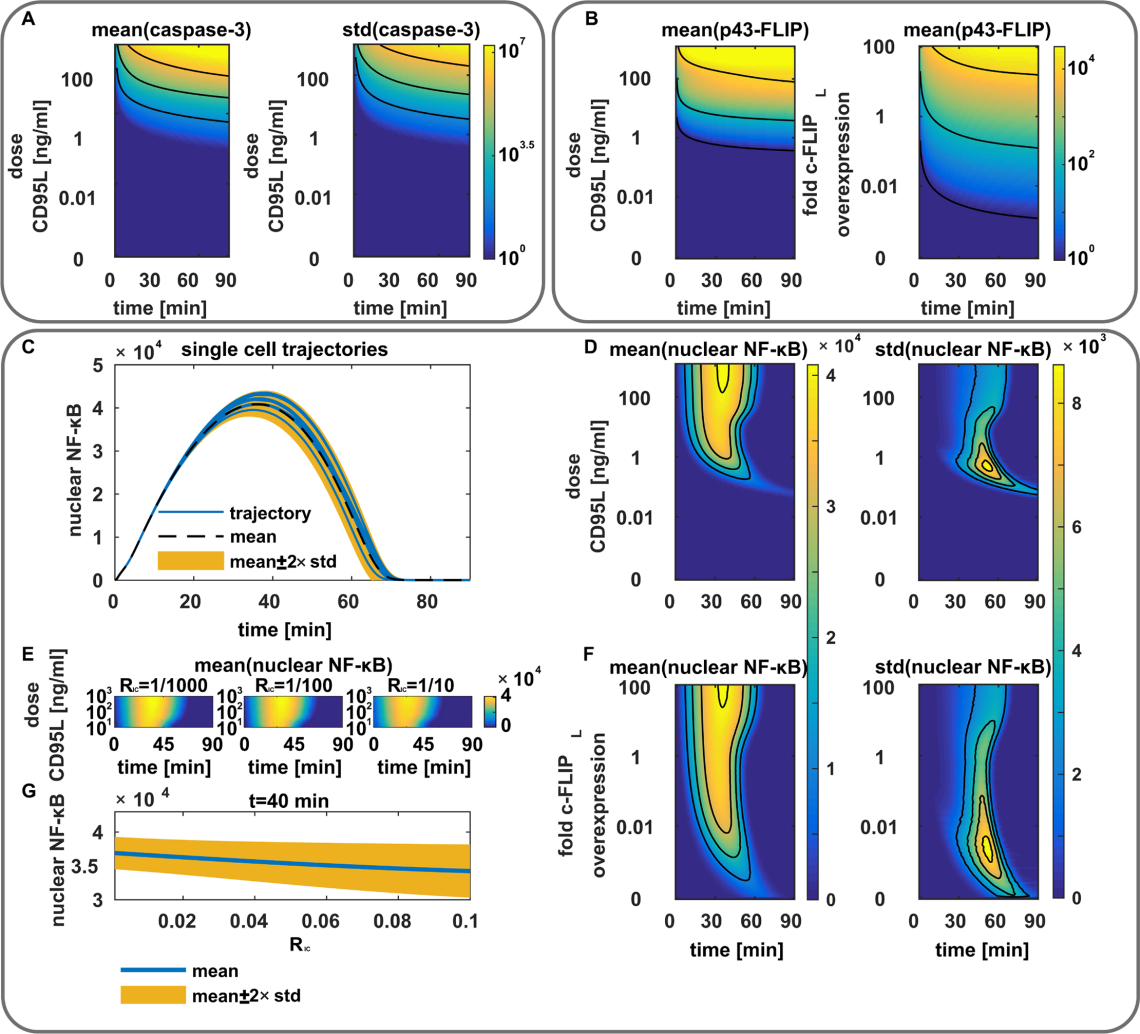
Analysis of computational model. (A) Model simulations of caspase-3 activation depending on the CD95L stimulation dose. Means and standard deviations (std) due to extrinsic noise are shown. (B) Model simulations for p43-FLIP generation depending on the c-FLIP_L_ expression levels and CD95L stimulation dose. (C) Subset of five single cell trajectories illustrating the abundance of nuclear NF-κB (blue) to the mean (black, dashed) and standard deviations (yellow). (D) Model prediction for the activation of nuclear NF-κB depending on the stimulation dose. Means and standard deviations are shown. (E) Analysis of the impact of different initial conditions of nuclear NF-κB (1/1000, 1/100, 1/10 of the total cellular amount of NF-κB) on the temporal dynamics. (F) Model prediction for the activation of nuclear NF-κB depending on the c-FLIP_L_ expression level. Means and standard deviations are shown. (G) Model prediction for the abundance of nuclear NF-κB (blue) for the time point 40 minutes depending on the initial conditions of nuclear NF-κB. Means (blue) and standard deviations (yellow) are shown. For all panels: model predictions for protein amounts are displayed in molecule numbers. The stimulation dose was set to 10 ng/ml if not indicated otherwise. For ease of interpretation only intrinsic noise was considered if extrinsic noise was not indicated.

To delineate the major features of the generated modeling network, we performed a detailed *in silico* analysis of central nodes of the model. The *in silico* investigation of the dependency of caspase-3 activation on the stimulation dose yields a two-dimensional landscape. In this landscape, upon increase of stimulation strength, means and standard deviations (SDs) of the active caspase-3 amounts rise exponentially (Fig 4A). According to the model, the rise in SD occurs due to the extrinsic noise in the initial abundances of procaspase-8 and c-FLIP. The extrinsic noise leads to a high variability in DED chain configurations and consequently active caspase-8 amounts, resulting in different levels of active caspase-3 generated within distinct cells. For the high stimulation strength above 100 ng/ml CD95L a high level of active caspase-3 was predicted, which is in line with experimental observations by us and others [24,35]. Hence, model simulation indicates that in the range of 10 to 100 ng/ml CD95L the amount of active caspase-3 strongly depends on the stimulation dose, which is highly variable from cell to cell due to the extrinsic noise (Fig 4A).

The *in silico* analysis of p43-FLIP generation resulted in a rise of means and SDs in p43-FLIP for increasing stimulation dose and/or expression levels of c-FLIP_L_ (Fig 4B, S7A Fig). p43-FLIP is generated in DED chains, which implies that its generation is influenced by extrinsic noise. It turned out that consideration of extrinsic noise *in silico* did not alter the mean value of p43-FLIP generation (S7B Fig). Instead extrinsic noise resulted solely in a spreading of the p43-FLIP distribution highlighted by an increased SD (S7B Fig). Modeling predicted a relatively fast processing of c-FLIP_L_ to p43-FLIP, accompanied by fluctuations of p43-FLIP with significant contributions of intrinsic and extrinsic noise. This is in accordance with a number of experimental data on fast processing of c-FLIP_L_ to p43-FLIP in the DED chain [19,20,23,36]. The next question was how this behavior would influence the dynamics of NF-κB activation.

Intriguingly, simulated time evolutions of NF-κB translocation to the nucleus have demonstrated a rather identical pattern for broad range of CD95L doses (Fig 4C). In particular, the detailed dose-dependent analysis of CD95-induced NF-κB translocation to the nucleus determined several regions of NF-κB response to CD95 stimulation *in silico* (Fig 4D). For stimulation doses between 0.5 and 5 ng/ml CD95L NF-κB activation took place, but there was a huge SD, while for stimulation doses above 5 ng/ml the activation was very robust against intrinsic noise, which is visualized by stable temporal patterns of means and SDs in a broad interval of stimulation strength (Fig 4D). Intriguingly, in contrast to several NF-κB activation pathways [37–39], different initial levels of nuclear p65 influence NF-κB activation did not show any dependence on NF-κB activation according to the analysis *in silico* (Fig 4E–4G, S7C Fig). The dependency of NF-κB activation on the initial concentration of c-FLIP_L_ at the DISC showed that for c-FLIP_L_ concentrations below the endogenous level an activation of NF-κB took place, but it was characterized by a high level of noise due to the low abundance of nuclear NF-κB (Fig 4F). For higher initial concentrations stable patterns of NF-κB activation were observed (Fig 4F). Further, the analysis of the extrinsic variability of the DISC leading to p43-FLIP generation and consequently NF-κB activation, has demonstrated that extrinsic noise does not significantly influence the p65 translocation dynamics (S7D Fig).

Modeling uncovered a very intriguing feature of CD95-mediated NF-κB activation: a stable pattern of activation, which is not dependent on the stimulation dose. In this way, CD95-mediated NF-κB induction is fundamentally different from a number of other NF-κB activation pathways. Hence, CD95-induced apoptosis and NF-κB have two opposite types of behavior: caspase-3 variability increases for all stimulation strengths, while NF-κB activation shows a robust behavior. This leads to the suggestion that starting from dosages above 5 ng/ml CD95L, heterogeneity in the apoptotic response should be dictated by the variability in the DED chain formation at the DISC rather than the stochastic gene expression in the NF-κB response. Next, we aimed on deriving a corresponding parameter, which can be used for predicting cell fate.

### The ratio of time of decision (TOD) vs. time of survival (TOS) as key parameter of cell fate

**Fig 5:**
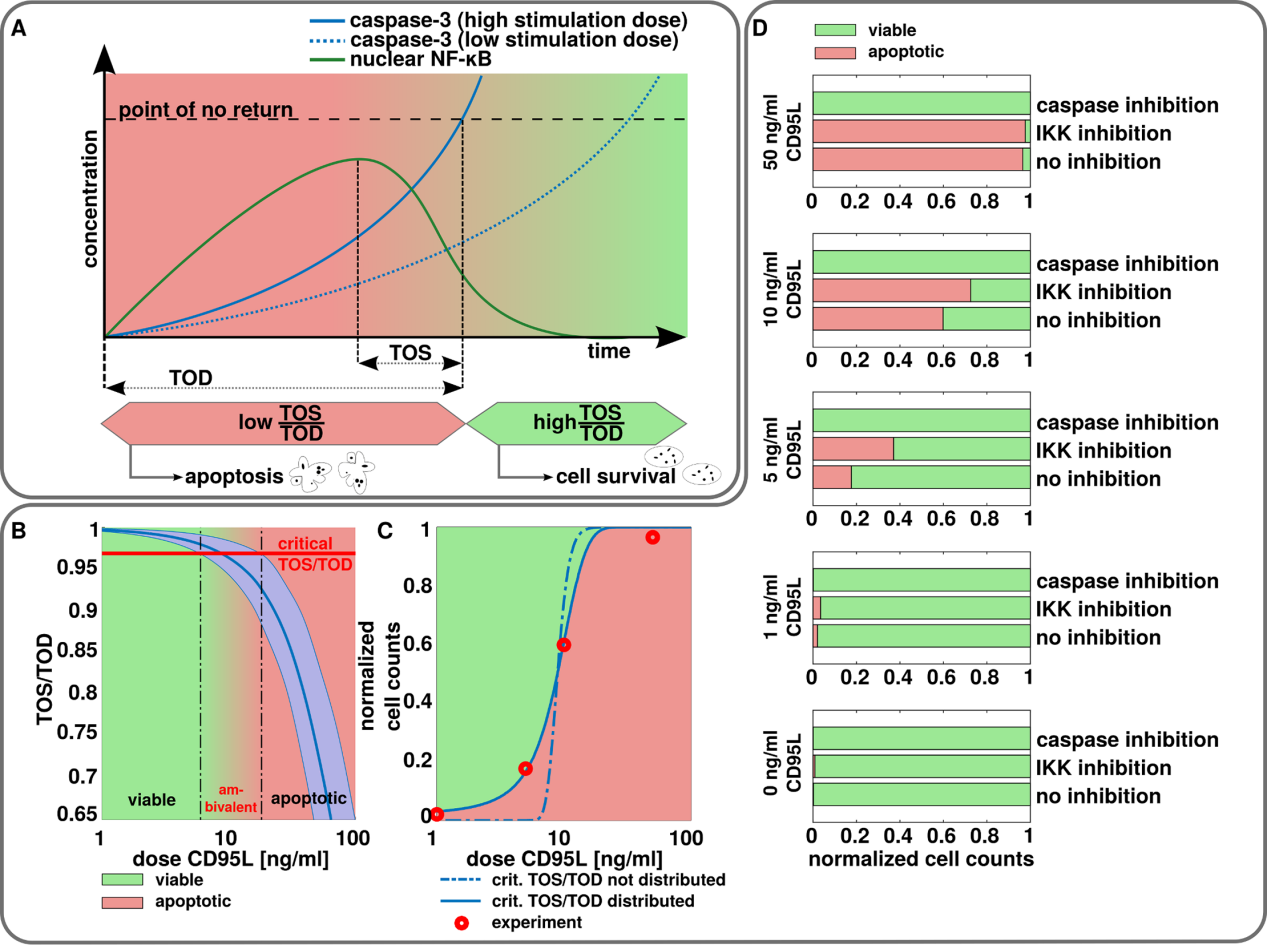
TOS/TOD mechanism and its validation. (A) Dynamics of NF-κB activation (green) and caspase-3 (blue). TOD (time of decision) is the time at which the concentration of active caspase-3 reaches ‘the point of no return’. TOS (time of survival) is the time interval between p65 translocation to the nucleus and when the point of no return is reached. (B) Model predictions for the temporal evolution of TOS/TOD. Mean (blue) and standard deviation (light blue) are shown. The mean of the critical TOS/TOD (r_crit_) is marked with a red line. (C) Normalized cell counts of viable (green) and apoptotic cells (red). Simulations of TOS/TOD *in silico* are shown by blue dashed line. When assuming a normal distributed critical TOS/TOD with a standard deviation of 1.5% of its mean the simulation fits well to the experimental data on cell death (blue, solid line). (D) Normalized cell counts regarding viable (green) and apoptotic (red) cells for indicated stimulation strength of CD95L and inhibition with zVAD-fmk or IKK inhibitor VII after 15 h. Cells were classified as viable or apoptotic as described in S1 Text. The measurements without inhibition are used to fit the dose-response curve in (C).

A cell undergoes apoptosis shortly after a critical concentration of caspase-3 is reached within a cell, which is known in the literature as the ‘point of no return’ [12,35,40]. The ‘point of no return’ was accordingly introduced into our model as an *in silico* value, which reflects the amount of active caspase-3, that is required for the particular cell to undergo apoptosis (Fig 5A). In order to estimate the critical amount of caspase-3 we used a quadratic discriminant analysis to discriminate between viable and apoptotic cells based on the caspase-3 fluorescence. This revealed a fluorescence threshold separating two subpopulations (S8 Fig). By linking the fluorescence to the abundance of caspase-3 estimated by our mathematical model we were able to compute the critical amount of caspase-3, which marks the ‘point of no return’. The time when the ‘point of no return’ is reached strongly depends on the stimulation dose (Fig 5A). NF-κB activation induces the transcription of genes that may counter the apoptotic pathway [41]. To delineate the connection between timing of NF-κB activation and apoptosis and thereby to get more insight into interplay between two pathways two new parameters were introduced: the time of decision (TOD), which is the time interval from stimulation to the ‘point of no return’ and the time of survival signaling (TOS), which is the interval from the maximum of NF-κB translocated to the nucleus to the ‘point of no return’ (Fig 5A). TOD and TOS are governed by the stimulation dose, since caspase-3 activation is sensitive to dose variation (Fig 5A). We hypothesized, that cells with a larger TOS/TOD ratio tend to survive the apoptotic stress, due to more time to counter the activation of the apoptotic machinery. In contrast, cells with small ratios fail to fight apoptosis due to the lack of time to synthesize anti-apoptotic proteins downstream of active NF-κB. For large doses the TOS/TOD ratio is negative, since the timing of the ‘point of no return’ precedes the timing of maximum nuclear NF-κB activation. In contrast, the TOS/TOD ratio tends to one for small stimulation doses since the maximum nuclear NF-κB activation occurs much earlier than the ‘point of no return’. Hence, TOS is approximately equal to TOD and their ratio yields one.

Next, we analyzed the effects of extrinsic noise from the DISC and intrinsic noise from NF-κB activation on the TOS/TOD ratio (Fig 5B, dark blue line). Both effects jointly led to a distributed TOS/TOD ratio (Fig 5B, blue region). In particular, NF-κB activation has its maximal variability from 0.5 to 5 ng/ml CD95L (Fig 4D, right panel), where TOS/TOD variance is negligibly small (Fig 5B). In the range of 5 to 10 ng/ml CD95L, NF-κB is activated but its variability is of minor importance (Fig 4D, right panel). On the other hand, variability in the abundance of the active caspase-3 starts to play a role already from very small CD95L stimulation doses and therefore has a major influence on the variability of TOS/TOD (Fig 5B). This *in silico* analysis pointed out that a response to CD95L stimulation between 5 and 10 ng/ml apparently presents a range of an ambivalent response, where single cells can either undergo apoptosis or survive upon the same stimulation strength.

To more closely relate the TOS/TOD ratio from our model to experimental data on apoptosis induction, we aimed to derive a critical ratio r_crit_ of TOS/TOD, which could predict cell fate in a single cell. A cell with TOS/TOD ratio > r_crit_ is very likely to survive, whereas a cell with TOS/TOD ratio < r_crit_ tends to undergo apoptosis. We expect r_crit_ to be positive so that the maximum of nuclear NF-κB activation occurs before the ‘point of no return’. In order to estimate the critical ratio we performed a least squares fit of the models using single cell simulations to match the dose-response curve from cell death data (Fig 5C, dashed line), which led to the value of r_crit_ = 0.965. The model qualitatively captures the sigmoid response, but there are discrepancies between theory and experiment. We believe that most likely this is due to additional biological variability, which was not accounted for in the model so far. If we assume that this variability causes slight variations of the critical TOS/TOD ratio in single cells we can fit our model almost perfectly to the experimental data, which strongly supports the validity of the TOS/TOD parameter for prediction of the life and death decisions (Fig 5C). By a least squares fit we obtained a relative SD of 1.5% of its mean. More statistics regarding the TOS/TOD ratio can be found in the S1 Text.

To further validate the TOS/TOD model we checked experimentally whether by influencing the TOS/TOD ratio apoptosis rates can be perturbed. For this purpose we used several CD95L stimulation strengths up to 50 ng/ml and longer stimulation periods of 15 hours in combination with inhibitors of caspases (zVAD-fmk) and NF-κB activation (IKK inhibitor VII). The distribution between living and apoptotic cells was analyzed via caspase-3 intensity and morphological features of the cells [42] (Fig 5D, S1 Text). The model predicted that the addition of the NF-κB inhibitor should significantly reduce TOS and thereby lead to a decrease of the TOS/TOD ratio compared to cells in which NF-κB is not inhibited. Addition of the IKK inhibitor indeed resulted in more apoptosis, which was detected upon low stimulation strengths of 5 ng/ml and 10 ng/ml (Fig 5D). This further confirmed that stimulation with CD95L at this dose range induces ambivalent response. Accordingly, the addition of caspase inhibitor increases the TOS/TOD ratio *via* increase of both TOS and TOD and therefore blocks apoptosis, which was subsequently observed in these experiments (Fig 5D). We also tested the effects of caspase and IKK inhibition using a different experimental approach based on live cell imaging and obtained similar results. Addition of IKK inhibitor VII resulted in a higher amount of apoptotic caspase 3/7 positive cells (S9 Fig, S1–S3 Videos). Importantly, the most sensitive region to the perturbations of TOS/TOD turned out to be the region between approximately 5 and 10 ng/ml, which further confirmed the model predictions.

Taken together this analysis uncovered three different regions of cell fate that can be identified with the TOS/TOD ratio: cell death, ambivalent response and cell survival, providing important insights into pathway dynamics and paves the way towards possible therapeutic applications.

## Discussion

A number of models addressing apoptosis and cell fate in single cells have been created [10,12,13]. However, single cell modeling of the interplay between apoptosis and anti-apoptosis pathways, which is a key feature of this model, has been missing so far. The understanding of this interplay at the single cell level is very important in the context of creation of efficient anti-cancer therapies specifically inducing apoptosis in every single cell. To understand the behavior of these competing pathways, we connected the strength of the apoptotic *vs*. NF-κB response with the stoichiometry of the DISC/DED chains using *in silico* analysis and single cell experimental data.

Most of the models describing single cell behavior use experimental data that are generated with artificially overexpressed caspase activity probes or tagged NF-κB proteins that might be influencing activation of this pathway. In contrast, our study is entirely focused on the analysis of endogenous proteins at the single cell level which is deemed possible due to the state of the art high throughput IFC analysis. Furthermore, IFC provides a platform to quantitatively assign single cell events over a large number of single cells using machine learning [42]. The latter potentially provides an enormous asset for building computational models and our study presents one of the first models of apoptosis and anti-apoptosis pathways implementing IFC data.

Among contemporary approaches there are several powerful single cell technologies that attract a major attention and allow to quantify endogenous proteins at the single cell level, in particular, IFC and single cell proteomics. Due to limited imaging channels in IFC this technology allows to follow only a limited number of proteins (up to 10) and is limited to the availability of suitable antibodies and dyes. In contrast to this, single cell proteomic approaches allow following a higher number of proteins. The advantage of IFC is that it allows following selected proteins. Therefore, in particular, with respect to quantitative biology and modeling signaling pathways IFC is undoubtedly the technology of choice.

In this study apoptosis and anti-apoptosis pathways in single cells upon DR stimulation were analyzed using a stochastic model. Strikingly, the model shows that NF-κB activation is highly variable around 1 ng/ml CD95L, whereas it becomes relatively robust at a higher CD95L stimulation strength starting from 5 ng/ml. In contrast, caspase-3 dynamics are highly variable for all dose ranges. Heterogeneity in caspase-3 activation is directly linked to the chain configuration at the DISC. The two distinct types of stochastic behavior of caspase-3 and NF-κB activation at a dose range of 5 to 10 ng/ml, lead to the fact that both scenarios of life or death are possible within this concentration range. This in turn creates a dose range of ‘ambivalent' response. Hence, we concluded that the major source of ambivalent response upon 5 to 10 ng/ml CD95L stimulation is heterogeneity in DED chain configuration, which results from extrinsic noise.

To more closely link our mathematical model of caspase-3 and NF-κB dynamics to experimental data, we introduced a parameter that determines the life/death decision in the cell and the threshold for apoptosis, the TOS/TOD ratio. Using this parameter could give a plausible explanation of the ultimate apoptotic response mechanism and its dose-dependent variability. In particular, the TOS/TOD ratio provides a clear explanation to the events occurring in the ambivalent response region between 5 and 10 ng/ml. In particular, even though both pathways are active upon stimulation above 5 ng/ml CD95L, cells can ‘fight’ apoptosis, resulting from the possibility of late activation of apoptotic downstream targets (TOS/TOD>r_crit_). For dosages around 5 ng/ml CD95L the chain configuration may in rare cases induce a quick response in caspase-3 (TOS/TOD<r_crit_) and the cell might undergo apoptosis (Fig 5B). Interestingly, if the TOS/TOD curve were steeper (Fig 5B), the ambivalent response range would decrease, whereas a more flat TOS/TOD curve increases the dose range of ambivalent response. We validated this behavior experimentally by inhibiting IKK and caspases, which fully confirmed TOS/TOD model predictions.

From our results we further conclude that on a single cell level, a life/death decision is very much dependent on the current state of the cell and the level of expression of anti-apoptotic *vs*. apoptotic proteins. For high stimulation strengths, the apoptotic decision prevails, whereas low stimulation strengths can also result in an anti-apoptotic response. The latter fits well to the recent findings by Roux et al. who have demonstrated that for low rates of DR-induced caspase-8 activity the cells might survive, which might be connected to the induction of the NF-κB pathway as we show in this study [12]. Here, we reason that this can be caused by dose/timing effects of two key signaling molecules related to life (NF-κB) and death (caspase-3). From our computational model we observed that activation of NF-κB is insensitive to stimulation dose variations above 5 ng/ml CD95L. This stems from the fact that NF-κB activation has a binary character owing to a time scale separation effect (signaling *vs*. gene expression time scale). Consequently, the stochastic gene expression related to NF-κB does not seem to be the main source of heterogeneity in the life/death decision. Rather, from our TOS/TOD ratio hypothesis, variations in the chain configuration and related caspase-3 fluctuations seem to be the dominant source of variability in the life/death response.

Defining the optimal strength of cell death induction leading to the eradication of cancer cells is a key step in anti-tumor therapy. The dynamics of cell death induction is often neglected despite its very important role in defining the life/death outcome. Furthermore, as we can conclude from analyzing the dynamics of CD95 signaling, the cross-talk between apoptotic and non-apoptotic pathways has to be considered for predicting the outcome of DR stimulation. The future challenge will be to define the dynamics of cell death responses in cancer *vs*. normal cells supported by powerful computational modeling in order to develop efficient anti-tumor therapies.

## Materials and methods

### Cell culture

Cervix carcinoma HeLa cell line with overexpression of CD95 (HeLa-CD95) [24,28] were cultivated in 5% CO_2_ at 37°C in DMEM/Hams F12 media (Biochrom, Berlin, Germany) with addition of 10% heat-inactivated FCS (Life Technologies, Darmstadt, Germany), 1% Penicillin/Streptomycin (Merck Millipore, Darmstadt, Germany) and 10 ng/ml Puromycin (Sigma Aldrich, Taufkirchen, Germany).

### Western blot

5*10^5^ HeLa CD95 cells were stimulated with CD95L for indicated time periods and concentrations [31]. After stimulation, cells were harvested by scraping, washed with PBS and lysed in lysis buffer (20 mM Tris HCl, pH 7.4, 137 mM NaCl, 2 mM EDTA, 10% glycerol, 1% Triton X-100, Protease Inhibitor mix (Roche, Mannheim, Germany)). 20 mg of protein lysate was separated on 12% stain-free SDS gels and blotted on nitrocellulose membrane (both Biorad, Hercules, USA). Membranes were blocked with 5% non-fat dried milk in PBS-T (0.05% Tween 20 in PBS) for one hour, washed three times with PBS-T and incubated with primary antibody overnight at 4°C. Before addition of the HRP-coupled isotype-specific secondary antibodies (Santa Cruz Biotechnology, Dallas, USA) the membrane was washed four times with PBS-T. After incubation the membrane was washed three times with PBS-T. HRP substrate (Luminata forte, Merck Millipore) was added and the chemiluminescence signals were captured with a ChemiDoc imaging system (Biorad). The images were quantified with the ImageLab 5.1 Software (Biorad). Special care was taken to avoid overexposure of images. Lanes and bands were selected manually and subsequently quantified. Anti-phospho-IκBα and anti-IκBα antibodies were purchased from Cell Signaling Technology (Danvers, USA). Anti-actin antibody was purchased from Sigma Aldrich (Taufkirchen, Germany). Anti-caspase-8 C15 and anti-c-FLIP antibodies were a kind gift of Peter H. Krammer (DKFZ, Heidelberg, Germany).

### Stimulation and staining of cells for flow cytometry

5*10^5^ HeLa cells were stimulated for indicated time periods with CD95L [33]. When indicated, cells were pre-stimulated with 50 μM zVAD-FMK (Bachem AG, Bubendorf, Switzerland) or 10 μM IKK inhibitor VII (MerckMillipore) for 30 minutes. After stimulation medium was removed, cells were washed with PBS and detached with trypsin-EDTA solution (Life Technologies, Darmstadt, Germany) for 5 minutes at 37°C. Afterwards, cells including medium, washing PBS, and trypsin were spun down at 500xg at 4°C for 5 minutes. Cell were fixed with 3% formaldehyde in PBS for 10 minutes at room temperature, permeabilized with 90% ice-cold methanol for 30 minutes on ice and washed twice with incubation buffer (5 g/l albumin fraction V (AppliChem, Darmstadt, Germany) in PBS). For antibody staining, cells were resuspended in 50 μl incubation buffer. Cells were stained for one hour in the dark with 1 μl anti-caspase-3 antibody, recognizing caspase-3 cleaved at Asp175, conjugated to AlexaFluor488 or AlexaFluor647, 0.5 μl anti-NF-κB-p65 antibody, conjugated to phycoerythrin (PE), (both purchased from Cell Signaling Technologies). After staining, cells were washed with incubation buffer and resuspended in 40 μl PBS. At least 5 minutes before measurement, 3 μl of the DNA dye 7AAD (BioLegend, San Diego, USA) was added.

### Imaging flow cytometry data acquisition and analysis

Imaging flow cytometry measurements were carried out using an Amnis FlowSight (Amnis/MerckMillipore). 10,000 events per sample were acquired using the channels 1 (bright field, 435–505 nm), 3 (560–595 nm), 5 (642–745 nm), 9 (bright field, 560–595 nm) and 11 (642–745 nm). The samples were excited with a 488 nm laser with 60 mW and a 642 nm laser with 100 mW laser power. Automated color compensation was performed with cells stained with one dye. Data were analyzed with IDEAS software version 6.1 (Amnis/ MerckMillipore). Single cells, with staining for 7AAD and p65 were selected and analyzed for p65 translocation [18]. Fluorescence intensity for antibody staining was analyzed with the intensity function of IDEAS.

### IncuCyte imaging analysis

On the day before stimulation 5*10^4^ HeLa-CD95 cells per well were seeded into a flat-bottom 96-well plate. Before stimulation media was replaced with 100 μl of media including IncuCyte Caspase-3/7 Apoptosis Assay Reagent (Essen Bioscience, Ann Arbor, USA) according to the manufacturer’s instructions. Then cells were incubated for 30 minutes with 10 μM IKK inhibitor VII (MerckMillipore) or 50 μM zVAD-FMK (Bachem AG) followed by CD95L stimulation. The first images were acquired 30 minutes after stimulation with IncuCyte ZOOM system (Essen Bioscience). Data analysis and videos were made with IncuCyte ZOOM software version 2015A1 (Essen Bioscience).

### Computational model

To perform single cell simulations, a mathematical model was developed and calibrated to experimental data. Since it was assumed that the formation of the DISC is several magnitudes faster than the downstream processes, the DISC formation was suggested to take place immediately after addition of CD95L. To account for the dose dependency of the DISC stoichiometry [20] the protein abundances of procaspase-8, c-FLIP_L_, and c-FLIP_S_ were modeled as dose dependent functions. We derived a working chain model by using data from Schleich et al. [20]. Although Schleich *et al*. used the SKW6.4 cell line and not Hela-CD95 cells, it was assumed that the same chain configuration prevails. Thus, the experimental data from [20] were extrapolated for this study. A complete list of species with initial conditions is provided in the S1 Text.

Biological systems often exhibit stochastic behavior, especially in gene networks with low copy numbers [43]. Therefore, the reactions accounting for the switching of genes between active and inactive states were modeled as stochastic processes and the remaining reactions deterministically. For simulation a hybrid version of the stochastic simulation algorithm [34] was used. Extrinsic variability was modeled as distributed initial conditions (including heterogeneous chain formations at the DISC), which were simulated with Monte Carlo sampling. Thus, we ended up with a hybrid stochastic-deterministic model. To efficiently simulate intrinsic and extrinsic noise during parameter optimization we used the sigma point method [44] combined with the hybrid stochastic simulation algorithm describing the interplay of distributed properties and low copy number effects at the gene expression level [45]. Since evolutionary algorithms haven been previously reported to yield excellent results when stochastic simulations are performed [42, 46] we used a genetic algorithm for optimization. For model calibration, western blot and flow cytometry data were used. Further details on the computational model and parameter optimization can be found in the S1 Text and S1 Appendix.

## Supporting information

S1 Text Modeling procedures. (PDF)

S1 Appendix Apoptosis model in SBML format. (XML)

S1 Table List of species used in the model topology. (EPS)

S2 Table Overview of reaction network. (EPS)

S1 Fig Analysis of CD95-induced caspase and NF-κB activation. (A) Scheme of the CD95/Fas signaling pathway, procaspase-8 and c-FLIP cleavage products. (B) HeLa-CD95 cells were stimulated with 250 ng/ml of CD95L for indicated time intervals with or without preincubation with zVAD-fmk; and subsequently analysed by western blot using the indicated antibodies. CD95 stimulation of HeLa-CD95 cells resulted in phosphorylation and degradation of IκBα occurring within 20 to 40 minutes after CD95 stimulation. This took place simultaneously with the appearance of p43-FLIP and p43/p41-procaspase-8 cleavage products as detected by western blot, followed by the appearance of the active caspase-3 subunit p17.
(PDF)

S2 Fig Imaging flow cytometry analysis of CD95 signaling in HeLa-CD95 cells. (A) Gating strategy for imaging flow cytometry experiments shown for stimulation of HeLa-CD95 cells with 250 ng/ml CD95L followed by staining with anti-p65 antibodies as well as of the nucleus with the DNA dye 7AAD. For subsequent analysis, focused images of single cells are selected. Similarity of the p65 and 7AAD signals in the nucleus serves as readout for NF-κB activation. (B) HeLa-CD95 cells were stimulated with 250 ng/ml CD95L for indicated times or with indicated doses of CD95L for 60 minutes. Cells were permeabilized and immunostained for p65, phospho-p65 and nucleus (7AAD) and analyzed with imaging flow cytometry for p65 translocation and p65 phosphorylation at Ser536. (C) Representative images of cells from experiment quantified in B.
(PDF)

S3 Fig The analysis of CD95 DISC formation in HeLa-CD95 cells. (A) HeLa-CD95 cells were stimulated with 250 ng/ml or 500 ng/ml CD95L for 20, 40 or 60 minutes. Cells lysates were used for immunoprecipitation (IP) with anti-APO-1 antibody. Cell lysates and IPs were analyzed with western blot and indicated antibodies. The right part of the figure is shown in the main text Fig 4A. (B) Independent repeat of the experiment from A.
(PDF)

S4 Fig Experimental western blot data used for the model calibration. HeLa-CD95 cells were stimulated with 250 ng/ml CD95L for indicated times. Western blot analysis was performed with the indicated antibodies, quantified and used for the calibration of the model.
(PDF)

S5 Fig Model calibration with the imaging flow cytometry data for NF-κB translocation to the nucleus. Experimental data (red) and simulations (blue) of NF-κB activation for HeLa-CD95 cells stimulated with indicated concentrations of CD95L and for indicated time intervals.
(PDF)

S6 Fig Model calibration with the imaging flow cytometry data for caspase-3 activation. Experimental data (red) and simulations (blue) of caspase-3 activation for HeLa-CD95 cells stimulated with indicated concentrations of CD95L and for indicated time intervals.
(PDF)

S7 Fig r Means and standard deviations of p43-FLIP and NF-κB. (A) Standard deviation of p43-FLIP corresponding to Fig 4B. (B) Means and standard deviations of p43-FLIP upon consideration of both intrinsic and extrinsic noises. (C) Investigation of the impact of different initial conditions of nuclear NF-κB (1/1000, 1/100, 1/10 of the total cellular amount of NF-κB in the nucleus on the temporal dynamics. (D) Means and standard deviations of NF-κB upon consideration of both intrinsic and extrinsic noise.
(PDF)

S8 Fig Estimating the critical amount of caspase-3. The distribution of viable (green, unstimulated) and apoptotic (red, 15h after stimulation with 50 ng/ml CD95L) cells regarding the caspase-3 fluorescence can be approximated by normal distributions, which differ in mean and variance. By applying a quadratic discriminant analysis the intersection point (black) can be calculated. For simplicity only a schematic illustration is provided.
(PDF)

S9 Fig Live cell imaging of HeLa-CD95 cells upon CD95L stimulation and addition of inhibitors of apoptosis and NF-kB pathways. (A) HeLa-CD95 cells were pre-incubated with 10 μM IKK inhibitor VII or 50 μM zVAD-fmk for 30 minutes and stimulated with 5 ng/ml CD95L for indicated time intervals. Caspase-3/7 activity was monitored with IncuCyte and IncuCyte Caspase-3/7 Apoptosis Assay Reagent. (A) shows the number of Caspase-3/7 positive cells per well. (B) shows representative pictures from (A). Cells that are positively stained for Caspase-3/7 activity can be observed in purple. Data from one out of two independent experiments measured as technical duplicates with four pictures per well are shown.
(PDF)

S10 Fig Sensitivity analysis of the TOS/TOD ratio. Sensitivity analysis of the TOS/TOD ratio in regard to the model rate constants (high stimulation doses). The rate constants are numbered according to S2 Table.
(EPS)

S11 Fig Sensitivity analysis of the TOS/TOD ratio. Sensitivity analysis of the TOS/TOD ratio in regard to the model rate constants (low stimulation doses). The rate constants are numbered according to S2 Table.
(EPS)

S12 Fig Confidence interval of the critical TOS/TOD ratio. The optimal value of r_crit_ is marked with a circle. Only r_crit_ values below the constant line are located in the 0.95 confidence interval.
(EPS)

S1 Video HeLa-CD95 cells were stimulated with 5 ng/ml CD95L for displayed time plus 30 minutes. Caspase-3/7 activity was monitored with IncuCyte and IncuCyte Caspase-3/7 Apoptosis Assay Reagent. Purple cells are positive for caspase-3/7 activity.
(AVI)

S2 Video HeLa-CD95 cells were pre-incubated with 10 μM IKK inhibitor VII for 30 minutes and stimulated with 5 ng/ml CD95L for displayed time plus 30 minutes. Caspase-3/7 activity was monitored with IncuCyte and IncuCyte Caspase-3/7 Apoptosis Assay Reagent. Purple cells are positive for caspase-3/7 activity.
(AVI)

S3 Video HeLa-CD95 cells were pre-incubated with 50 μM zVAD-fmk for 30 minutes and stimulated with 5 ng/ml CD95L for displayed time plus 30 minutes. Caspase-3/7 activity was monitored with IncuCyte and IncuCyte Caspase-3/7 Apoptosis Assay Reagent. Purple cells are positive for caspase-3/7 activity.
(AVI)
