# Supplementary material for: Quantitative single cell analysis uncovers the life/death decision in CD95 network
Source: PLoS Comput Biol. 2018 Sep 26;14(9):e1006368. doi: 10.1371/journal.pcbi.1006368 (PMC6175528; doi:10.1371/journal.pcbi.1006368)
Supplement: S1 Text — (PDF) [file pcbi.1006368.s001.pdf]

Supplementary Information

Quantitative single cell analysis uncovers the life/death decision in CD95 network

Jörn H. Buchbinder[1], Dennis Pischel[1], Kai Sundmacher[1, 2], Robert J. Flassig[2], and Inna N. Lavrik[1,3]

[1]Otto-von-Guericke-University, Universitätsplatz 2, D-39106 Magdeburg
[2]Max Planck Institute for Dynamics of Complex Technical Systems, Sandtorstraße 1, D-39106 Magdeburg
[3]Corresponding author contact: inna.lavrik@med.ovgu.de

July 9, 2018

Contents

1 Model Description **1**

2 Modeling **2**

3 Error Model for Protein Quantification **3**

4 Optimization **4**

5 Classification of Apoptotic and Viable Cells **4**

6 Further Analysis of the TOS/TOD Ratio **5**

1 Model Description

In this study we developed a stochastic two compartment model consisting of the cytosol and the nucleus, which is strongly inspired by the models used in (Fricker et al., 2010; Pękalski et al., 2013). For simplicity the formation of the death inducing signaling complex (DISC) was assumed to happen very fast. Based on (Schleich et al., 2012) the DISC was modeled with a chain topology. Although in (Schleich et al., 2012) a different

cell line (SKW6.4) was used it was assumed that the same chain configuration and the ratios at the DISC prevail. The dose dependent ratios were derived by extrapolation of the data from (Schleich et al., 2012) (see Fig. 3 in the main document). The mean values of protein abundances of caspase-8 and c-FLIP$_\mathrm{L}$ at the DISC were presumed to be proportional to the stimulation dose in accordance with (Schleich et al., 2012). The proportionality factor was treated as an unknown model parameter and identified during model calibration. The abundance of c-FLIP$_\mathrm{RS}$ at the DISC was considered to be proportional to c-FLIP$_\mathrm{L}$ with a proportionality factor derived from Suppl. Tab. 1 in (Fricker et al., 2010). A complete list of species with initial conditions is provided in Tab. 1.

Experimental data show that there is a large variability in the stoichiometry of the DISC within a cell population (Schleich et al., 2012). Thus, the extrinsic variability of the DISC was modeled with distributed initial conditions of its core components. We assumed that the proteins caspase-8, c-FLIP$_\mathrm{L}$ and c-FLIP$_\mathrm{RS}$ follow a log-normal distribution with a standard deviation proportional to the mean. We derived the proportionality factor from Suppl. Tab. 1 in (Fricker et al., 2010). The model used in this study contains two species of genes producing a negative regulator and IκBα. Both genes are capable of switching between an on and off state. Switching to the on state is triggered by nuclear NF-κB and switching to the off state is triggered by nuclear IκBα. The switching of the genes is modeled *via* a stochastic process capturing the probabilistic transitions. The topology of the whole model can be seen in Fig. 2 in the main document.

2 Modeling

In this study a stochastic model governed by the chemical master equation (CME)

$$\frac{\mathrm{d}}{\mathrm{d}t}P(\boldsymbol{x},t) = \sum_{k=1}^{m} a_k(\boldsymbol{x}-\boldsymbol{N}_k)P(\boldsymbol{x}-\boldsymbol{N}_k,t) - a_k(\boldsymbol{x})P(\boldsymbol{x},t). \tag{1}$$

was used. The solution of the CME yields the temporal evolution of the probability P to find the system in a certain state $\boldsymbol{x}$ at time point t. $\boldsymbol{N}$ denotes the stoichiometric matrix and $\boldsymbol{a}$ is the propensities, which represent the stochastic transition rates from one state to a neighbored one. The index k indicates the k's reaction. A list of reactions with the corresponding transition rates is provided in Tab. 2.

Our model accounts for two sources of variability, namely intrinsic noise due to probabilistic reactions and extrinsic noise due to cell-to-cell variability. In this study the proteins procaspase-8, c-FLIP$_\mathrm{L}$ and c-FLIP$_\mathrm{RS}$ are considered to have log-normally distributed initial conditions (Tab. 1). For the remaining chemical species no information regarding the initial distribution was available. Thus, deterministic initial conditions were assumed.

The most popular techniques to tackle these issues are the Gillespie algorithm (Gillespie, 2007) to simulate the probabilistic reactions and Monte Carlo (MC) sampling of the distributed initial conditions. Since both methods are computational very demanding

an approximate technique developed in a previous study is used to make the model calibration feasible (Pischel et al., 2017). This technique combines the Sigma Point (SP) method (Julier et al., 2000) with an approximate version of the Gillespie algorithm (Haseltine and Rawlings, 2002). Instead of following single cell trajectories, as done in conventional MC sampling, our approximate technique relies on the computation of the stochastic temporal dynamics of cell populations. Thereby we compute for every time point of interest 1000 probability density functions, which depict a random variable, since we used a stochastic process for the temporal evolution. The superposition of these densities yields a robust approximate solution of the CME. For more information see (Pischel et al., 2017).

3 Error Model for Protein Quantification

To calibrate the mathematical model the simulation results were compared to the experimental data derived from western blot and imaging flow cytometry measurements. For the western blot data a multiplicative error model was used, which led to log-normally distributed measurement errors (Kreutz et al., 2007)

$$M_{WB} \quad = \quad M_{0,WB} + \alpha_{WB} x \varepsilon_{WB}. \tag{2}$$

The offset $M_{0,WB}$ was derived by Eq. 2 to the initial time point. α_{WB} is a constant identified during model calibration and ε_{WB} measurement noise $\varepsilon_{WB} \sim e^{\mathcal{N}(0,\sigma^2)}$. The parameter σ characterizing the spread of the noise was also identified during model calibration. In this case x denotes the population mean of a specific protein.
For the imaging flow cytometry data the following error model for protein quantification was developed

$$M_{Flow} \quad = \quad M_{0,Flow} + \alpha_{Flow} x (1 + \varepsilon_{Flow}). \tag{3}$$

In this case the measured signal M_{Flow}, $M_{0,Flow}$, and x represent a probability distribution. The proportionality constant α was identified during model calibration. For caspase-3 the measured signal is given by the fluorescence intensity of the caspese-3 anti-body, whereas for NF-κB the similarity score was used

$$M = 10 + \ln\left(\frac{1+p}{1-p}\right). \tag{4}$$

Here p denotes the Pearson correlation coefficient. A large similarity value indicates a high abundance of total nuclear NF-κB and a low similarity a low abundance. For caspase-3 and NF-κB $M_{0,Flow}$ was determined from the initial time point $t = 0\,\text{min}$. The measurement noise was normally distributed with the standard deviation proportional to the mean. The proportionality constant was determined during model calibration.

4 Optimization

To estimate the unknown parameters a least squares optimization approach was used. The objective function

$$\hat{F} = \sum_{i,j} \frac{(\hat{M}_{exp,i,j} - \hat{M}_{sim,i,j})^2}{\hat{\sigma}^2_{i,j}} \tag{5}$$

was minimized to find a set of optimal parameters. $\hat{M}_{exp,i,j}$ are the experimental measurement signals and $\hat{M}_{sim,i,j}$ the simulated responses. The sum includes all measured proteins i and time points j. $\hat{\sigma}$ represents the standard deviation of the measurements. The standard deviation was computed from experimental data. Technical replicates were measured for the western blot measurements . If no repeated measurements were performed, as for imaging flow cytometry data, the standard deviation was set to 1. For the western blots $\hat{M}_{exp}$ represents the normalized gray values on log-scale obtained by experiments and $\hat{M}_{sim}$ the simulated ones. For the flow cytometry measurements $\hat{M}_{exp} - \hat{M}_{sim}$ represents the euclidean distance between the experimental and simulated probability distribution, see (Cha, 2007) for a detailed overview of distance measures.
We chose a genetic algorithm with a population size of 200 individuals and 10^4 generations as stochastic optimizer. It was assumed that after the optimization the set of parameters was very close to the global optimum. Supplementary Fig. 5-6 and Fig. 3 in the main document show the comparison between the experimental measurements and simulation results of the western blot and flow cytometry data.
In order to quantify the goodness of fit we used the reduced χ^2-statistics χ^2/ν. Here χ^2 denotes the χ^2 statistics and ν the degrees of freedom, which can be calculated from the number of observations N and the number of fitted parameters q using

$$\nu = N - q - 1. \tag{6}$$

With $\chi^2 = 69.7$, $N = 39$ and $q = 64$ we obtain a value for the reduced χ^2 statistics of 2.9. As rule of thumb the model fits the experimental data well if χ^2/ν is close 1. Hence, no significant over or underfitting can be observed.

5 Classification of Apoptotic and Viable Cells

To identify viable and dead cells measured by imaging flow cytometry a quadratic discriminant analysis was used. This machine learning algorithm has to be trained with features containing discriminative information of the experimental data and the corresponding class label c (dead or alive) for each cell. The features used in this study are intensity of caspase-3 and the area threshold (area of the nucleus). From previous studies we know that for the characterization of stationary distributions these features are very suited Pischel et al. (2018); Schmidt et al. (2015). To train the algorithm a data set without stimulation and a data set with sufficient time after the stimulation was used. It was assumed that the cells from the first data set were all alive and the cells from the second data set were all dead. The algorithm fits a multivariate normal distribution F_k

for each class derived by the empirical class mean and covariance to the training data and predicts the class label K of unseen data f by maximizing the posterior probability P_{post}

$$K = \arg\max_k P_{post}(c = k | F_k = f). \tag{7}$$

The ratios of viable and apoptotic cells shown in Fig. 5C,D were obtained by this method.

6 Further Analysis of the TOS/TOD Ratio

In oder to complete our investigation regarding the TOS/TOD ratio we performed a sensitivity analysis, see Suppl. Fig. 10-11. Therefore the rate constants k were varied from $\frac{1}{10}\,k$ to $10\,k$. Additionally we computed the confidence interval for critical TOS/TOD ratio r_{crit}, see Suppl. Fig. 12. The optimal r_{crit} was derived by minimizing the sum of least squared residuals $S(r_{crit})$. To estimate the confidence interval we checked for which values of r_{crit} the following condition holds true

$$\frac{S(r_{crit}) - S(r^*_{crit})}{S(r^*_{crit})} \leq \frac{q}{N-q} F(\alpha, q, N-q). \tag{8}$$

In our case $q = 1$ denotes the number of parameters, $N = 5$ the number of observations, $\alpha = 0.05$ the significance level and r^*_{crit} the optimal value of the critical TOS/TOD ratio. The 0.95 confidence intervall ranges from 0.96 to 0.97, see Suppl. Fig. 12.

References

Sung-Hyuk Cha. Comprehensive survey on distance/similarity measures between probability density functions. *International Journal of Mathematical Models and Methods in Applied Sciences*, 1:300–307, 2007. doi: 10.1186/1471-2105-8-376.

N. Fricker, J. Beaudouin, P. Richter, R. Eils, P.H. Krammer, and I.N. Lavrik. Model-based dissection of CD95 signaling dynamics reveals both a pro- and antiapoptotic role of c-FLIP$_\text{l}$. *Journal of Cell Biology*, 190(3):377–389, 2010. doi: 10.1083/jcb.201002060.

D.T. Gillespie. Stochastic simulation of chemical kinetics. *Annual Review of Physical Chemistry*, 58:35–55, 2007. doi: 10.1146/annurev.physchem.58.032806.104637.

E.L. Haseltine and J.B. Rawlings. Approximate simulation of coupled fast and slow reactions for stochastic chemical kinetics. *Journal of Chemical Physics*, 117(15):6959–6969, 2002. doi: 10.1063/1.1505860.

S. Julier, J. Uhlmann, and H.F. Durrant-Whyte. A new method for the nonlinear transformation of means and covariances in filters and estimators. *IEEE Transactions on Automatic Control*, 45(3):477–482, 2000. doi: 10.1109/9.847726.

C. Kreutz, M.M.B. Rodriguez, T. Maiwald, M. Seidl, H.E. Blum, L. Mohr, and J. Timmer. An error model for protein quantification. *Bioinformatics*, 23(20):2747–2753, 2007. doi: 10.1093/bioinformatics/btm397.

J. Pękalski, P.J. Zuk, M. Kochańczyk, M. Junkin, R. Kellogg, S. Tay, and T. Lipniacki. Spontaneous NF-κB activation by autocrine TNFα signaling: A computational analysis. *PLoS ONE*, 8(11), 2013. doi: 10.1371/journal.pone.0078887.

D. Pischel, K. Sundmacher, and R.J. Flassig. Efficient simulation of intrinsic, extrinsic and external noise in biochemical systems. *Bioinformatics*, 33(14):i319–i324, 2017. doi: 10.1093/bioinformatics/btx253.

D. Pischel, J.H. Buchbinder, K. Sundmacher, I.N. Lavrik, and R.J. Flassig. A guide to automated apoptosis detection: How to make sense of imaging flow cytometry data. *PLoS ONE*, 13(5), 2018. doi: 10.1371/journal.pone.0197208.

K. Schleich, U. Warnken, N. Fricker, S. Öztürk, P. Richter, K. Kammerer, M. Schnlzer, P.H. Krammer, and I.N. Lavrik. Stoichiometry of the CD95 death-inducing signaling complex: Experimental and modeling evidence for a death effector domain chain model. *Molecular Cell*, 47(2):306–319, 2012. doi: 10.1016/j.molcel.2012.05.006.

J.H. Schmidt, S. Pietkiewicz, M. Naumann, and I.N. Lavrik. Quantification of CD95-induced apoptosis and NF-κB activation at the single cell level. *Journal of Immunological Methods*, 423:12–17, 2015. doi: 10.1016/j.jim.2015.04.026.
