# Supplementary figures and images for: Quantitative single cell analysis uncovers the life/death decision in CD95 network
Source: PLoS Comput Biol. 2018 Sep 26;14(9):e1006368. doi: 10.1371/journal.pcbi.1006368 (PMC6175528; doi:10.1371/journal.pcbi.1006368)

A

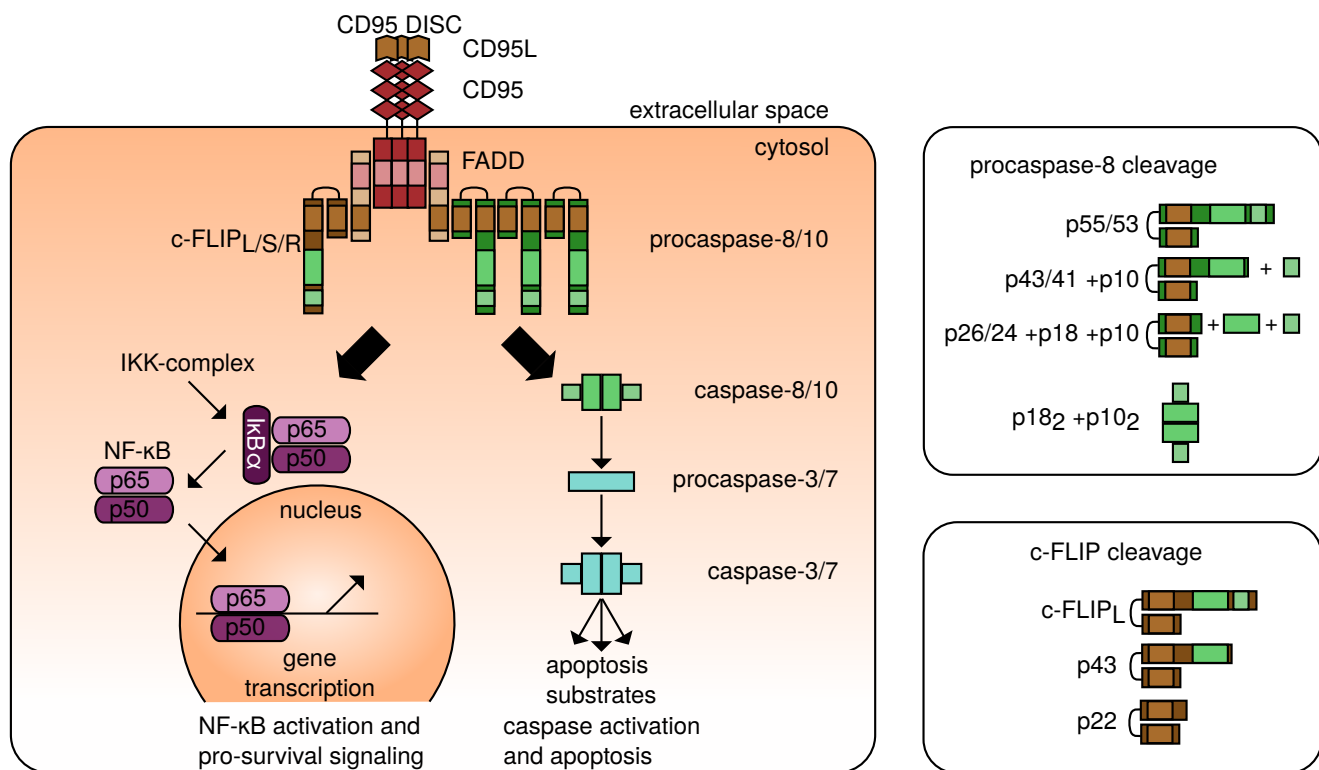

B

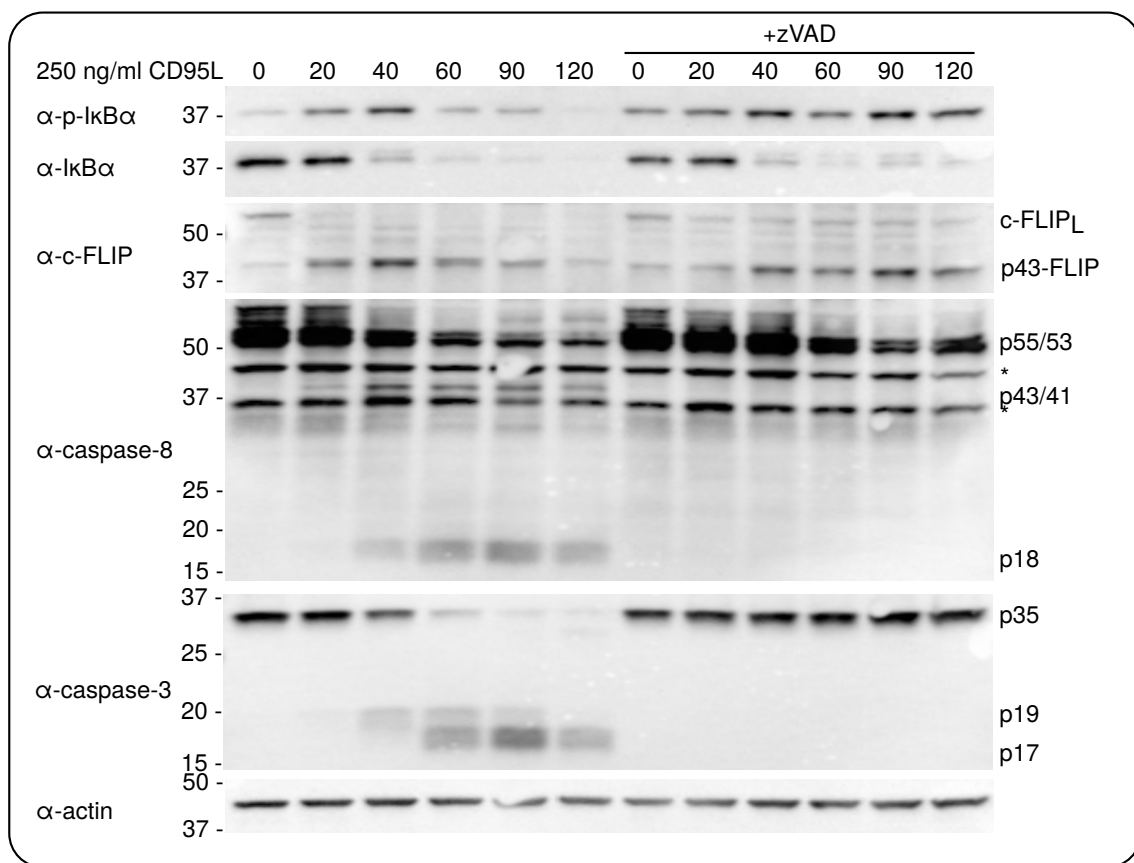

Supplement: S1 Fig — (A) Scheme of the CD95/Fas signaling pathway, procaspase-8 and c-FLIP cleavage products. (B) HeLa-CD95 cells were stimulated with 250 ng/ml of CD95L for indicated time intervals with or without preincubation with zVAD-fmk; and subsequently analysed by western blot using the indicated antibodies. CD95 stimulation of HeLa-CD95 cells resulted in phosphorylation and degradation of IκBα occurring within 20 to 40 minutes after CD95 stimulation. This took place simultaneously with the appearance of p43-FLIP and p43/p41-procaspase-8 cleavage products as detected by western blot, followed by the appearance of the active caspase-3 subunit p17. (PDF) [file pcbi.1006368.s005.pdf]

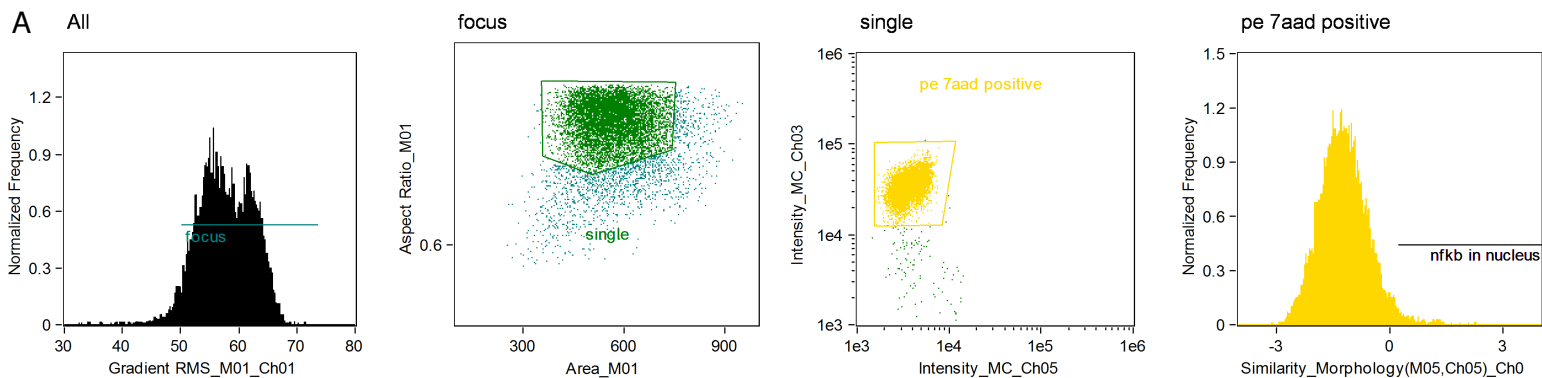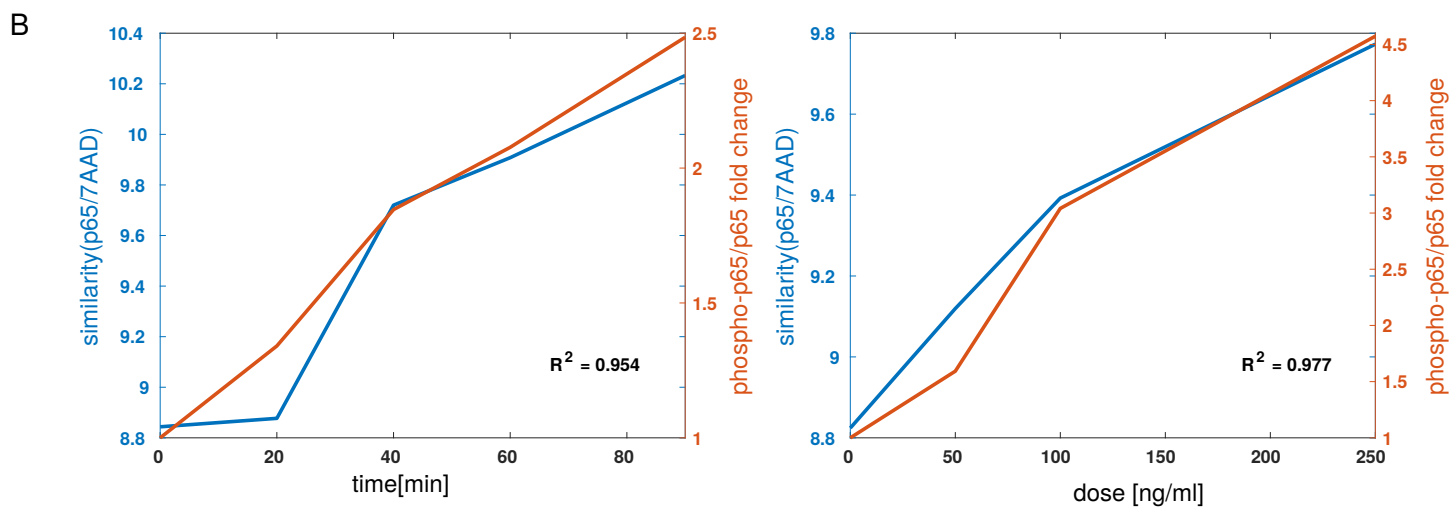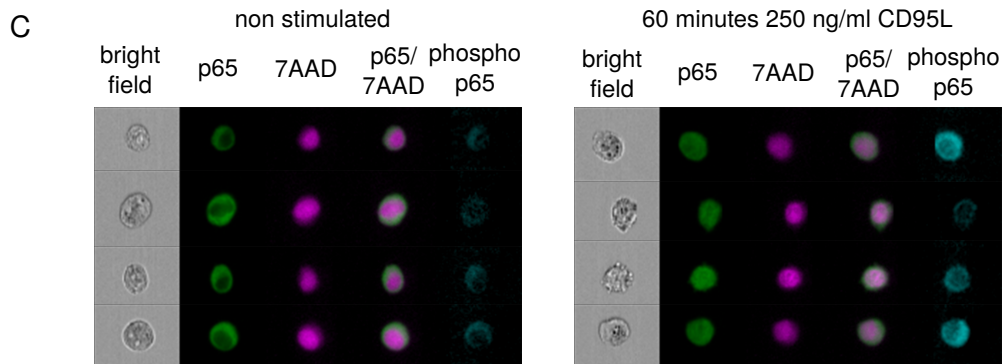

Supplement: S2 Fig — (A) Gating strategy for imaging flow cytometry experiments shown for stimulation of HeLa-CD95 cells with 250 ng/ml CD95L followed by staining with anti-p65 antibodies as well as of the nucleus with the DNA dye 7AAD. For subsequent analysis, focused images of single cells are selected. Similarity of the p65 and 7AAD signals in the nucleus serves as readout for NF-κB activation. (B) HeLa-CD95 cells were stimulated with 250 ng/ml CD95L for indicated times or with indicated doses of CD95L for 60 minutes. Cells were permeabilized and immunostained for p65, phospho-p65 and nucleus (7AAD) and analyzed with imaging flow cytometry for p65 translocation and p65 phosphorylation at Ser536. (C) Representative images of cells from experiment quantified in B. (PDF) [file pcbi.1006368.s006.pdf]

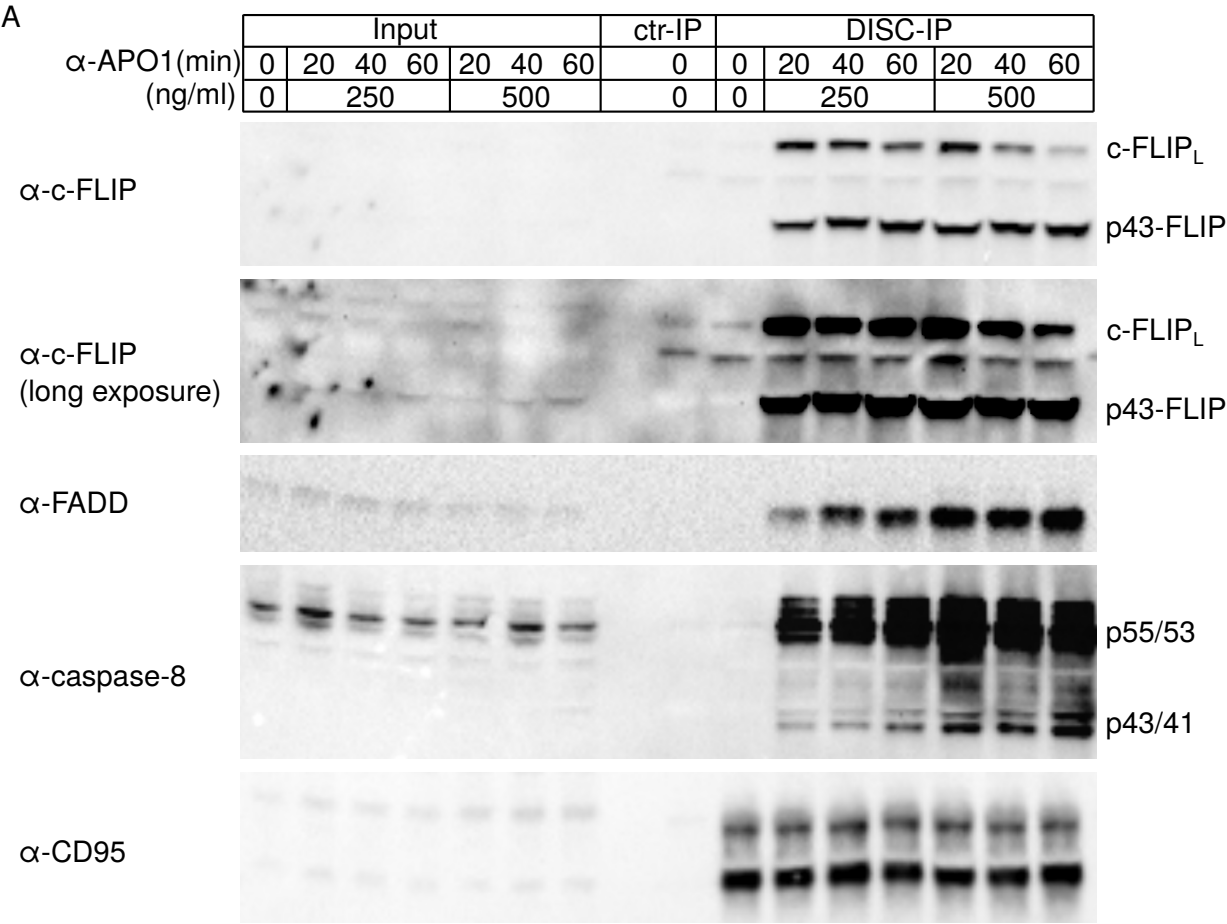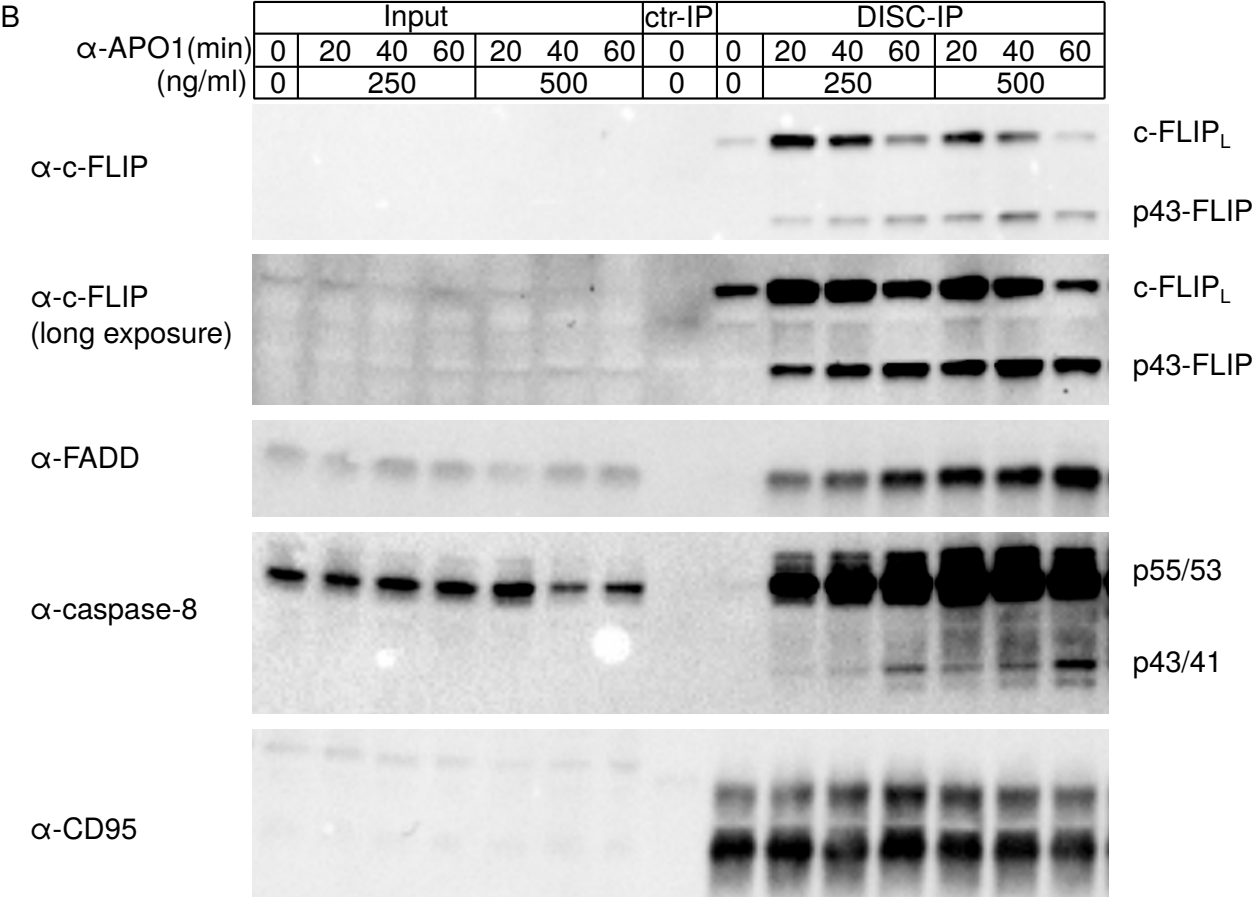

Supplement: S3 Fig — (A) HeLa-CD95 cells were stimulated with 250 ng/ml or 500 ng/ml CD95L for 20, 40 or 60 minutes. Cells lysates were used for immunoprecipitation (IP) with anti-APO-1 antibody. Cell lysates and IPs were analyzed with western blot and indicated antibodies. The right part of the figure is shown in the main text Fig 4A. (B) Independent repeat of the experiment from A. (PDF) [file pcbi.1006368.s007.pdf]

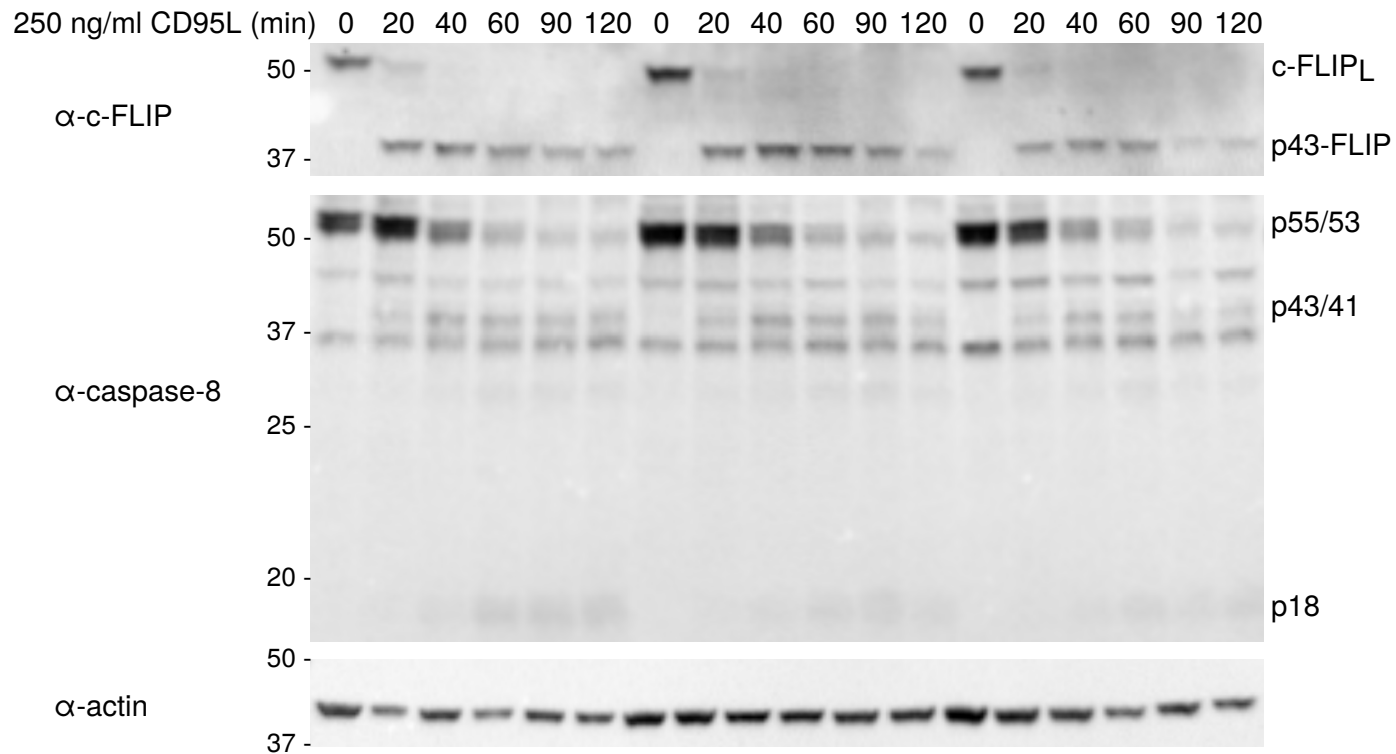

Supplement: S4 Fig — HeLa-CD95 cells were stimulated with 250 ng/ml CD95L for indicated times. Western blot analysis was performed with the indicated antibodies, quantified and used for the calibration of the model. (PDF) [file pcbi.1006368.s008.pdf]

— Simulation  
— Experiment

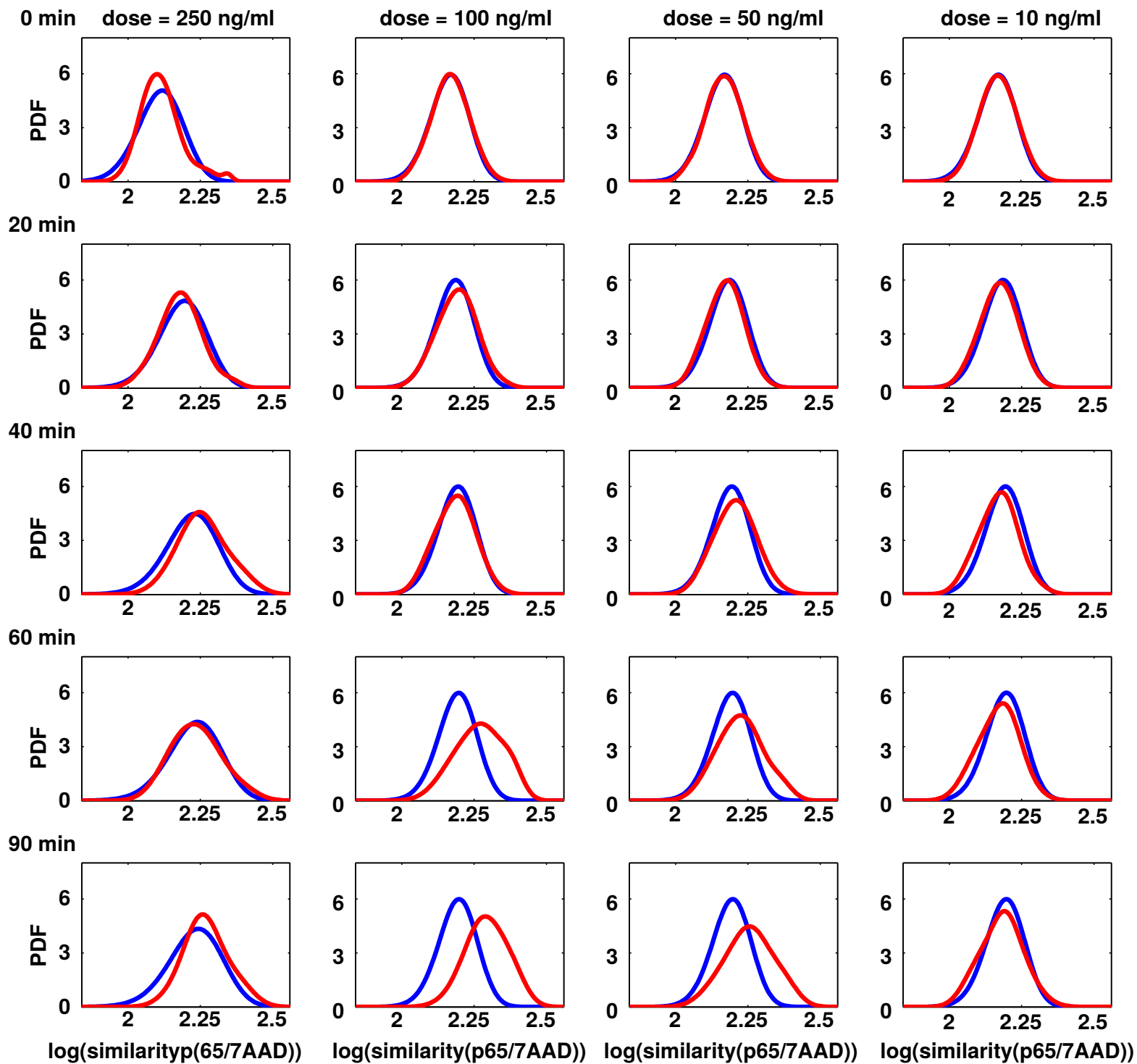

Supplement: S5 Fig — Experimental data (red) and simulations (blue) of NF-κB activation for HeLa-CD95 cells stimulated with indicated concentrations of CD95L and for indicated time intervals. (PDF) [file pcbi.1006368.s009.pdf]

— Simulation  
— Experiment

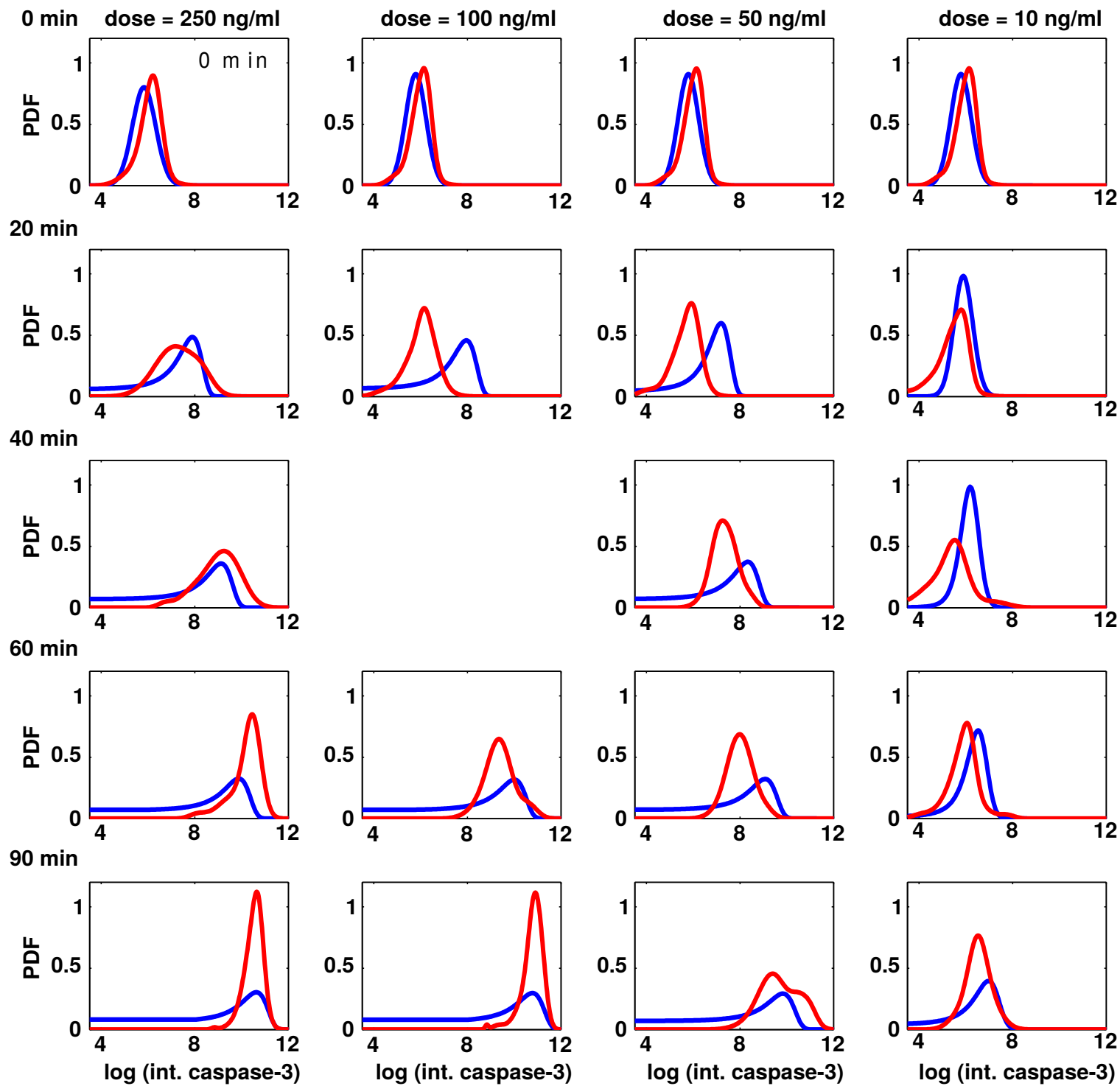

Supplement: S6 Fig — Experimental data (red) and simulations (blue) of caspase-3 activation for HeLa-CD95 cells stimulated with indicated concentrations of CD95L and for indicated time intervals. (PDF) [file pcbi.1006368.s010.pdf]

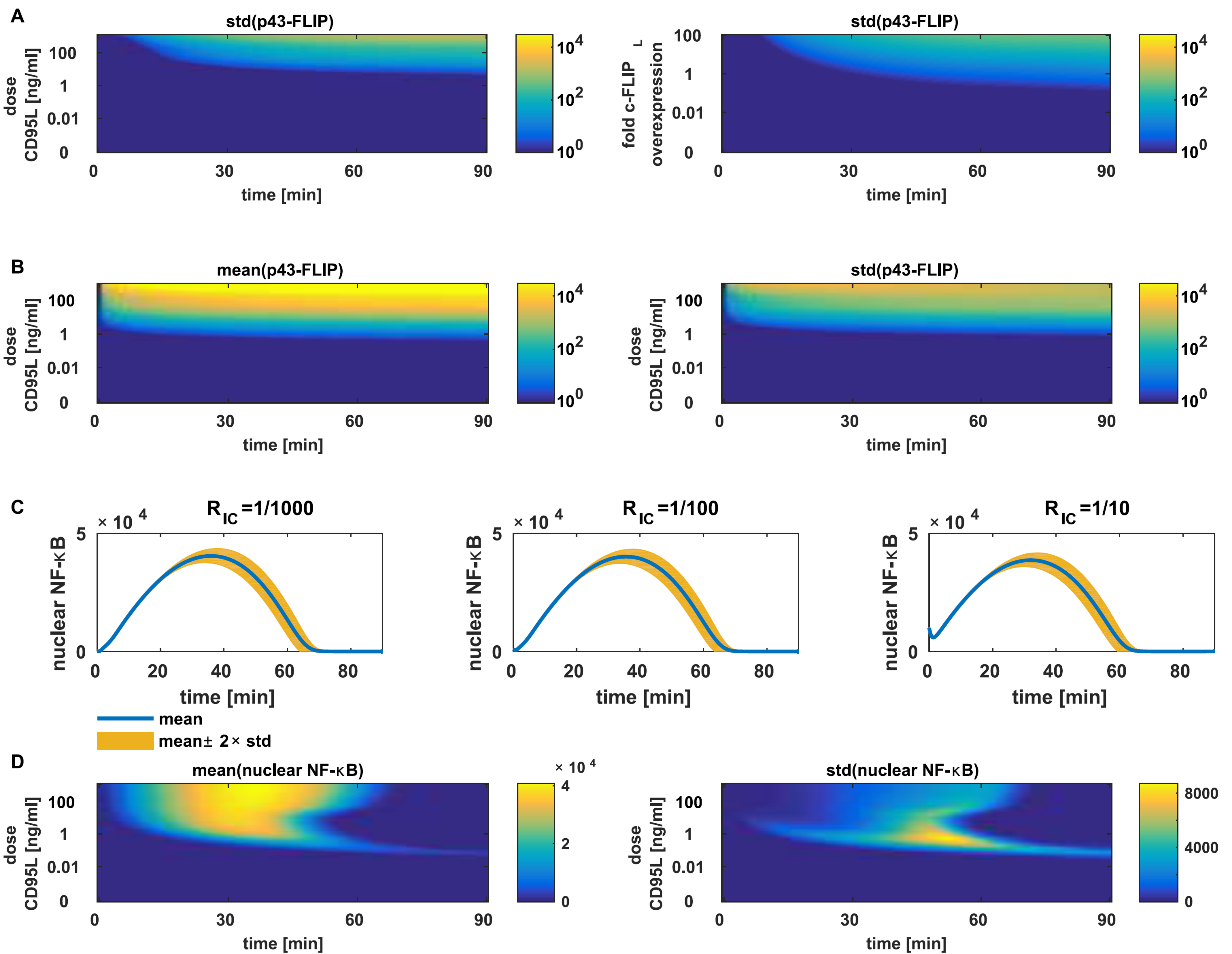

Supplement: S7 Fig — (A) Standard deviation of p43-FLIP corresponding to Fig 4B. (B) Means and standard deviations of p43-FLIP upon consideration of both intrinsic and extrinsic noises. (C) Investigation of the impact of different initial conditions of nuclear NF-κB (1/1000, 1/100, 1/10 of the total cellular amount of NF-κB in the nucleus on the temporal dynamics. (D) Means and standard deviations of NF-κB upon consideration of both intrinsic and extrinsic noise. (PDF) [file pcbi.1006368.s011.pdf]

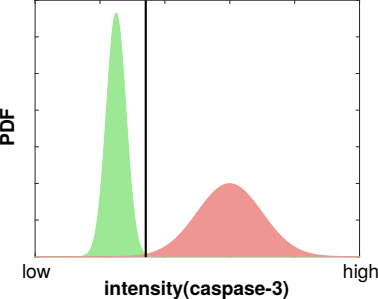

Supplement: S8 Fig — The distribution of viable (green, unstimulated) and apoptotic (red, 15h after stimulation with 50 ng/ml CD95L) cells regarding the caspase-3 fluorescence can be approximated by normal distributions, which differ in mean and variance. By applying a quadratic discriminant analysis the intersection point (black) can be calculated. For simplicity only a schematic illustration is provided. (PDF) [file pcbi.1006368.s012.pdf]

A

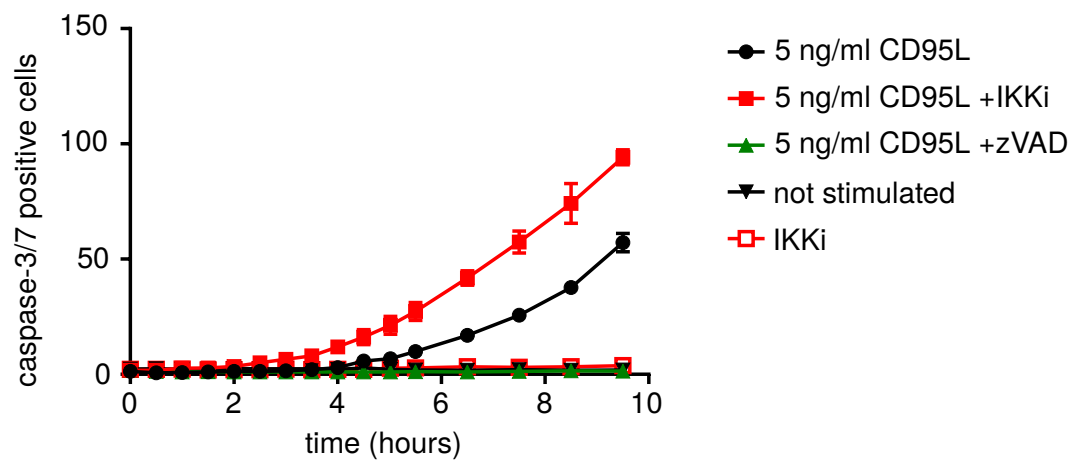

B

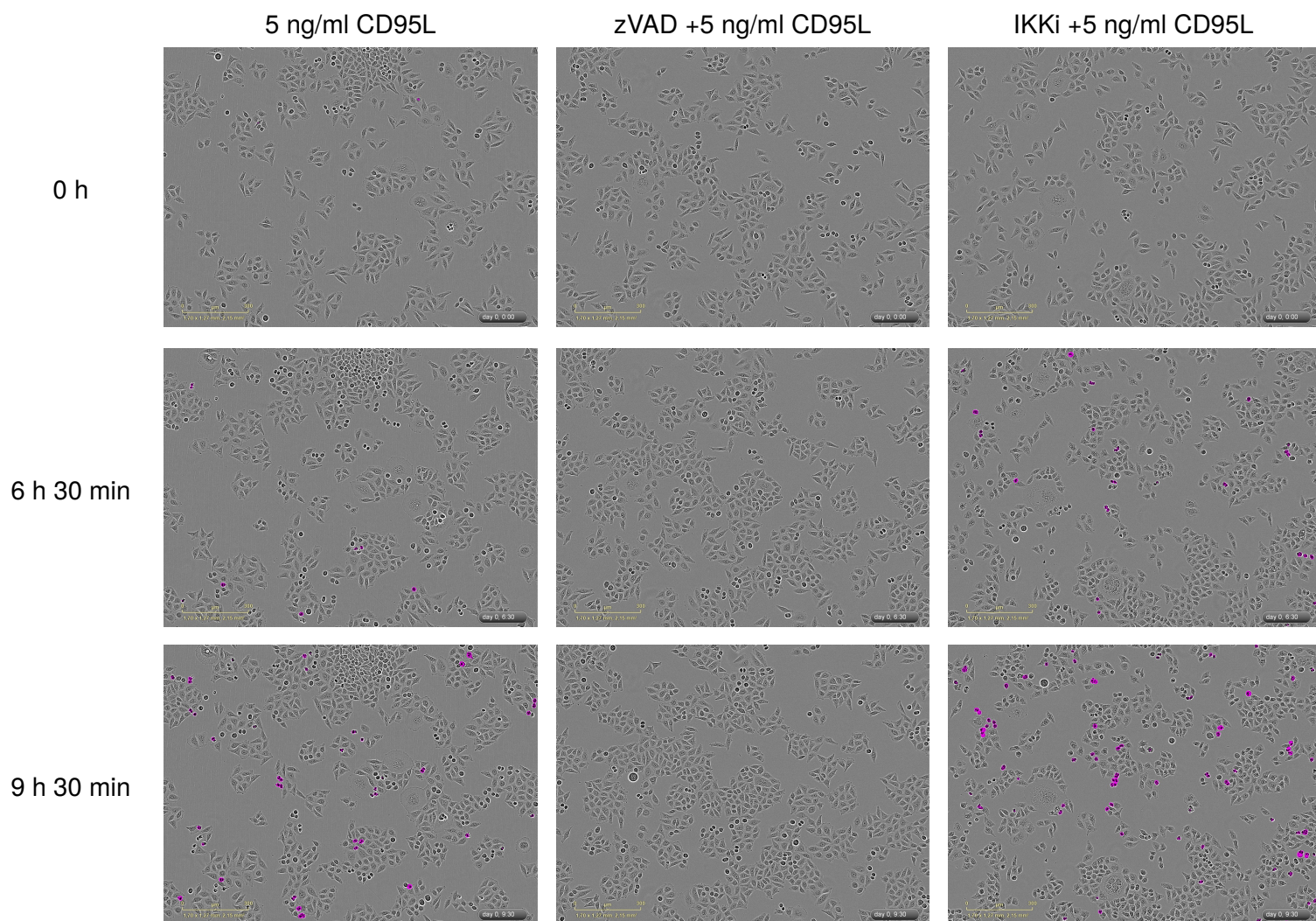

Supplement: S9 Fig — (A) HeLa-CD95 cells were pre-incubated with 10 μM IKK inhibitor VII or 50 μM zVAD-fmk for 30 minutes and stimulated with 5 ng/ml CD95L for indicated time intervals. Caspase-3/7 activity was monitored with IncuCyte and IncuCyte Caspase-3/7 Apoptosis Assay Reagent. (A) shows the number of Caspase-3/7 positive cells per well. (B) shows representative pictures from (A). Cells that are positively stained for Caspase-3/7 activity can be observed in purple. Data from one out of two independent experiments measured as technical duplicates with four pictures per well are shown. (PDF) [file pcbi.1006368.s013.pdf]
